# Spatial Patterns of Leaf Carbon, Nitrogen, and Phosphorus Stoichiometry of Aquatic Macrophytes in the Arid Zone of Northwestern China

**DOI:** 10.3389/fpls.2018.01398

**Published:** 2018-09-21

**Authors:** Xusheng Gong, Zhiyan Xu, Wei Lu, Yuqing Tian, Yaheng Liu, Zhengxiang Wang, Can Dai, Jinghui Zhao, Zhongqiang Li

**Affiliations:** Hubei Collaborative Innovation Center for Green Transformation of Bio-Resources, Hubei Key Laboratory of Regional Development and Environmental Response, Faculty of Resources and Environment, College of Life Sciences, Hubei University, Wuhan, China

**Keywords:** aquatic macrophytes, arid zone, ecological stoichiometry, environmental factors, temperature-plant physiology hypothesis

## Abstract

Ecological stoichiometry is a powerful indicator for understanding the adaptation of plants to environment. However, understanding of stoichiometric characteristics of leaf carbon (C%), nitrogen (N%), and phosphorus (P%) for aquatic macrophytes remains limited. In this study, 707 samples from 146 sites were collected to study the variations in leaf C%, N%, and P%, and tried to explore how different environmental conditions affect leaf C, N, and P stoichiometry. Results showed that the mean values of leaf C%, N%, P%, and N:P ratios were 39.95%, 2.12%, 0.14%, and 16.60% of macrophytes across the arid zone of northwestern China, respectively. And the mean values of leaf P% were lower than those from the Tibetan Plateau and eastern China, which maybe due to an adaptation strategy of the plants to the unique conditions in the arid zone in the long-term evolutionary process. The higher N:P ratios suggested that P was established as the limiting factor of the macrophytes communities in the arid zone of northwestern China. There were significant differences in leaf C%, N%, P%, and their ratios among different life forms. Our results also showed strong relationships between leaf N% and N:P ratios and longitude, leaf N%, P%, and N:P ratios and latitude, and leaf N% and P% and altitude, respectively. In addition, the results showed that pH can significantly influence leaf C%. Our results supported the temperature-plant physiology hypothesis owing to a negative relationship between leaf N% and P% of macrophytes and mean annual temperature in the arid zone of northwestern China. The different patterns of leaf stoichiometry between the arid zone of northwestern China and eastern China indicated that there were different physiological and ecological adaptability of macrophytes to environmental gradients in different climatic zones.

## Introduction

Ecological stoichiometry is a fundamental discipline that studies the balance between energy and various chemical elements in biological interactions and nutrient cycling in ecosystems (Elser et al., 2000, 2003, 2010). Such research not only helps to distinguish between different regional ecological stoichiometry patterns (Han et al., 2005), but also better determines the relationship between the pattern and environmental variables (Han et al., 2011). In addition, this research can help to characterize the biogeochemical cycles (Hedin, 2004; Reich and Oleksyn, 2004; Portillo-Estrada et al., 2013).

In the last twenty years, many studies are focused on large-scale plant leaf stoichiometry and demonstrated that leaf N% and P% increased with increasing latitude (Mc Groddy et al., 2004; Reich and Oleksyn, 2004; Han et al., 2005; Wang et al., 2011) and altitude (Körner, 1989). However, other studies indicated that the leaf N% and P% decreased with altitude (Soethe et al., 2008; Zhao et al., 2014). These inconsistencies suggest that many crucial research questions of the leaf stoichiometry patterns and determinants have not been thoroughly elucidated to date. Moreover, most studies of plant leaf stoichiometry have focused on terrestrial ecosystems, and considerably less attention has been devoted to freshwater systems. Until recently, only a small number of studies have examined regional geographical patterns of leaf stoichiometry in some freshwater macrophytes (Xia et al., 2014; Li et al., 2015; Wang et al., 2015), and the results of these studies indicated that variability in foliar N%, P%, and N:P stoichiometry across diverse habitats showed considerable differences (Xia et al., 2014; Li et al., 2015; Wang et al., 2015). These different results suggested that geographical patterns of leaf stoichiometry of macrophytes may change with regard to the spatial extent of the study and geographical location of the study area and highlighted that further studies are needed to understand geographical patterns of leaf stoichiometry at different spatial scales and study areas.

Leaf nutrient contents and stoichiometry are sensitive to such factors as spatial scale, habitats, and plant types (Li et al., 2010; Van de Waal et al., 2010; Wu et al., 2012). Previous studies showed that the leaf N:P ratio changed with soil age gradient (Reich and Oleksyn, 2004), precipitation, and temperature (He et al., 2006, 2008). Further studies showed that the main factors shaping leaf nutrient stoichiometry varied with spatial scale and study area (He et al., 2006, 2008). To date, many hypotheses have been proposed (Thompson et al., 1997; Tjoelker et al., 1999; Weih and Karlsson, 2001; Sterner and Elser, 2002; Elser et al., 2003). Among the hypotheses, Temperature-Biogeochemistry Hypothesis (TBH) and Temperature-Plant Physiology Hypothesis (TPPH) are generally accepted by biogeographers and ecologists. TBH proposed that lower temperatures lead to the low activity of microbes, which reduces soil N and P, resulting in the insufficient leaf N and P of plants (Aerts and Chapin, 1999). TPPH assumed that plants from habitat of low temperature and high latitude may have a higher N and P content in their leaves to counterbalance the depressed biochemical efficiency caused by low temperatures (Oleksyn et al., 1998; Weih and Karlsson, 2001). However, these studies have largely been conducted on terrestrial plants, hindering further examination of the mechanistic basis of large-scale patterns of leaf stoichiometry in freshwater systems (Su et al., 2016). Thus, further research on leaf nutrient contents and stoichiometry of aquatic macrophytes is necessary (Li et al., 2015).

There were three climate zones in China, including a humid monsoon climate zone in eastern China, a cold area of the Tibetan plateau, and an arid zone in northwestern China (Feng et al., 1989; Tang et al., 1992). Recently, the leaf stoichiometry pattern of aquatic macrophytes was studied in eastern China (Xia et al., 2014) and the Tibetan plateau (Wang et al., 2015), which indicated that the variability in foliar N%, P%, and N:P stoichiometry and the main factor affecting the leaf stoichiometry pattern in each climate zone showed considerable differences. In the arid zone of northwestern China, extreme aridity gradients exist over relatively short distances in both the west-east and north–south directions (Feng et al., 1989; Tang et al., 1992). Over such environmental transects, plants encounter a variety of microclimates differing in temperature, precipitation and vapor pressure gradients, each of which may influence the variation of plant nutrient stoichiometry. In this study, we collected 707 aquatic plant samples from 146 sampling sites across the arid area of northwestern China, including 92 species (56 species of emergent plants, 7 species of floating-leaved plants, 29 species of submerged plants) from 37 genera and 24 families. The leaf ecological stoichiometry patterns of aquatic macrophytes were analyzed to investigate the relationships between environmental factors and plant leaf element contents. Two major questions were stressed: (1) what is the leaf stoichiometry pattern of the aquatic macrophytes and their general causes in the arid zone of northwestern China? (2) what are the differences of leaf stoichiometry patterns of the aquatic macrophytes and their main environmental factors among the three climatic zones in China?

## Materials and Methods

### Study Area

**FIGURE 1:**
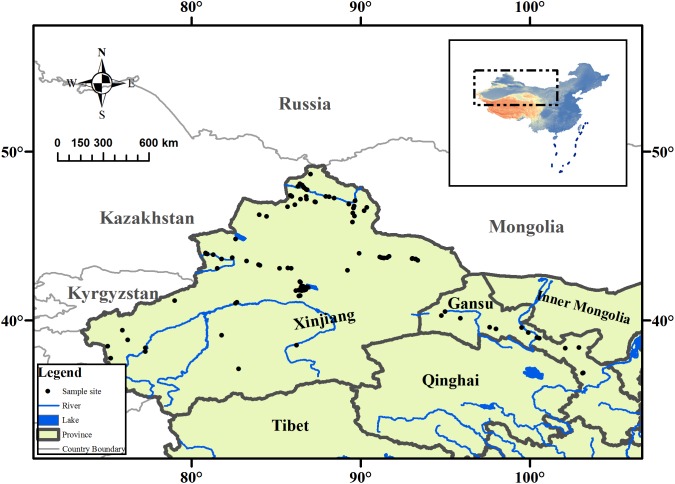
Map showing the collection sites of aquatic plant, and its location in the arid zone of northwestern China.

The arid zone (35°–49°N, 73°–106°E) is a land-locked region located in northwestern China (**Figure 1**) and is surrounded by the Qinghai-Tibet Plateau and many high mountains. The climate is generally water limited, and steppe biomes are prevalent. The annual rainfall in the arid zone is less than 250 mm, with certain areas receiving less than 100 mm annually, but the annual evaporative capability is above 2000 mm. The mean annual temperature is 2–6°C, with the maximum monthly mean temperature being above 28°C, and the minimum monthly mean temperature is below −16°C; and the daily temperature fluctuates significantly (Feng et al., 1989).

### Sampling and Measurements

The aquatic macrophyte collections were conducted in the arid zone from July to October in 2011. The field sites in this study covered almost entire the arid zone of northwestern China, which ranged from 36° to 55°N, 80° to 103°E, and the altitude varied from 79 m to 3,185 m above sea level (**Figure 1**). At each sampling site, latitude, longitude, and altitude were recorded using the global positioning system (GPS). We also measured the pH of each water body using a portable pH meter. Mean annual temperature (MAT) and mean annual precipitation (MAP) were obtained by entering geographic coordinates into equations derived from data collected at meteorological stations across China (Fang et al., 2001). The model formula is given by

MMT(MP)=a×Latitude+b×Longitude+c×Altitude+d

where a, b, c, and d are the regression coefficients.

According to estimated monthly mean temperature and monthly precipitation, MAT and MAP of each site were calculated.

Plants collected at each site were placed in paper envelopes and dried in the sun. These samples were dried to a constant mass at 60° for 72 h in the oven upon returning to the laboratory. All dried leaf samples of each plant were ground to a fine powder with a mortar in the laboratory. The total C and N contents were determined with an elemental analyser (NA2500, Carlo Erba Reagenti, Milan, Italy). The total P was measured using a sulfuric acid/hydrogen peroxide digest and the ammonium molybdate ascorbic acid method (Sparks et al., 1996). The detailed data is shown in **Appendix S1**.

### Data Analysis

SPSS Statistics 19 was used for statistical analysis. We calculated the mean and standard deviation (SD) of leaf C%, N%, P%, C:N, C:P, and N:P ratios for all species and each life form. Analysis of variance was applied to determine the statistical significance of the differences of leaf C%, N%, P%, C:N, C:P, and N:P ratios from different life forms (*p* < 0.05). Pearson correlations among the leaf C%, N%, P%, C:N, C:P, and N:P ratios were also calculated for all observations. Before performing one-way ANOVA, all data were tested for normality and homogeneity. Non-normal data were transformed (log10) to obtain normality. We used leaf C%, N%, P%, C:N, C:P, and N:P ratios to perform linear regressions with longitude, latitude and altitude. We included the effects of fixed (MAT, mean annual temperature; MAP, mean annual precipitation, pH and Life forms) and random (Species nested in Plot) on leaf C%, N%, P%, C:N, C:P, and N:P ratios to build linear mixed effect model. Linear mixed effect model was also made for each life forms. Redundancy analyses (RDA) were also performed with CANOCO for windows (version 5) to elucidate the relationship between plant C:N:P signatures and the environmental parameters (**Appendix S2**).

## Results

### Pattern and Variation of Leaf C%, N%, P% Contents and Their Ratios in the Arid Zone of Northwestern China

For all species and sites pooled (observations *n* = 707), foliar C%, N%, P%, and C:N, C:P, N:P of aquatic macrophytes across the study areas varied widely. The mean of leaf C%, N%, and P% of aquatic macrophytes across the arid zone of northwestern China was 39.95%, 2.12%, and 0.14%, respectively, which varied from 19.67% to 47.79%, 0.22% to 5.45%, and 0.03% to 1.15%. The mean of C:N, C:P, N:P ratios was 23.55, 346.91, and 16.60, respectively, with a range of 7.29 to 187.41 for the C:N ratio, 37.32 to 1256.81 for the C:P ratio, and 2.55 to 55.09 for the N:P ratio (**Table 1**).

**Table 1:** Leaf C%, N%, P%, and their ratios for overall observations and different life forms. Different letters indicated significant differences in leaf C%, N%, P%, and their ratios between different life forms.

Plant group	Overall	Life form			Sig.
		Submerged	Floating-leaved	Emergent	
n	707	220	29	458	
C%	39.95 ± 4.60	36.48 ± 5.48b	40.11 ± 4.81a	41.61 ± 2.90a	<0.001
N%	2.12 ± 0.91	2.05 ± 0.79b	2.66 ± 0.93a	2.12 ± 0.92b	0.004
P%	0.14 ± 0.08	0.15 ± 0.07b	0.20 ± 0.15a	0.14 ± 0.08b	0.001
C:N	23.55 ± 15.96	20.54 ± 8.58b	16.84 ± 5.89b	25.43 ± 18.58a	<0.001
C:P	346.91 ± 176.29	296.08 ± 134.53b	252.03 ± 115.95b	377.34 ± 189.13a	<0.001
N:P	16.60 ± 7.85	14.99 ± 5.16b	15.93 ± 7.06ab	17.41 ± 7.85a	<0.001

*The number of samples (n) and mean values (%) (±SD) are shown. Different letters indicate significant differences at p < 0.05. Sig, significance level.*

In the arid zone of northwestern China, submerged plants had the lowest leaf C%, and sharply lower than that of emergent plants and floating-leaved plants. For the leaf N% and P%, floating-leaved plants presented significantly higher than the other two life forms. Our results showed there were significant differences in the leaf C:N, C:P, and N:P ratios among the three life forms, and emergent plants presented the highest leaf C:N, C:P, and N:P ratios (**Table 1**).

### Relationships Between the Leaf C%, N%, P%, and Their Ratios

Correlation analyses indicated that the leaf C%, N%, P%, and the C:N C:P and N:P ratios were correlated with each other (**Table 2**). The leaf C% showed strong positive correlations with the leaf N% and the C:N, C:P, N:P ratios, but it was not related to the leaf P%. Leaf N% was strongly negatively correlated with C:N and C:P ratios and strongly positively correlated with leaf P% and N:P ratio. The leaf P% presented strong negative correlations with C:N, C:P, and N:P ratios. The C:N ratio displayed strong negative correlations with N:P ratio, and positive correlations with C:P ratios. Additionally, strong positive correlations were observed between C:P and N:P ratios.

**Table 2:** Pearson correlation among leaf element contents.

	C%	N%	P%	C:N	C:P	N:P
C%	1.00	0.107^∗∗^	0.003	0.101^∗∗^	0.255^∗∗^	0.155^∗∗^
N%		1.00	0.500^∗∗^	−0.962^∗∗^	−0.436^∗∗^	0.488^∗∗^
P%			1.00	−0.500^∗∗^	−0.967^∗∗^	−0.493^∗∗^
C:N				1.00	0.500^∗∗^	−0.468^∗∗^
C:P					1.00	0.524^∗∗^
N:P						1.00

*^∗∗^p < 0.01, ^∗^p < 0.05.*

### Relationships Between the Leaf C%, N%, P%, and Their Ratios and Environmental Factors

**FIGURE 2:**
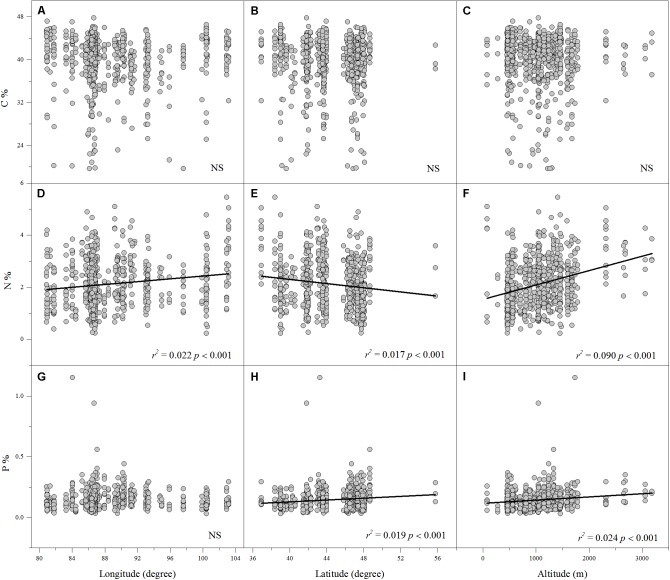
The relationships between leaf C%, N% and P% and longitude, latitude and altitude **(A–I)**. (Sig. *p* < 0.05).

**FIGURE 3:**
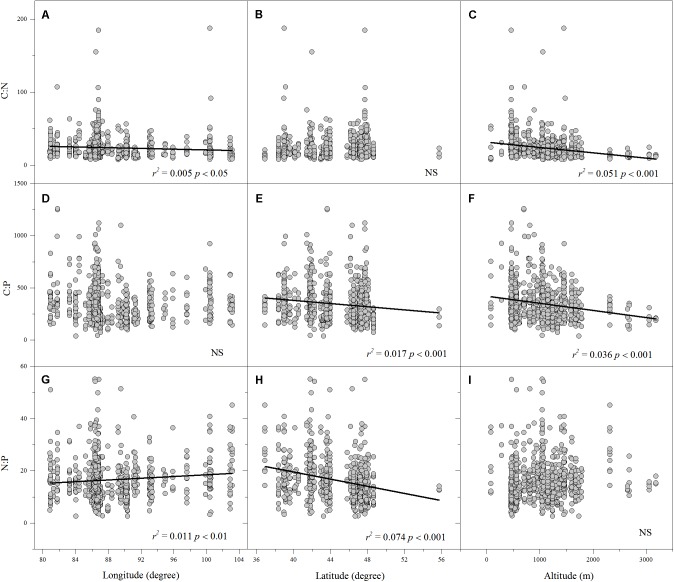
The relationships between leaf C:N, C:P and N:P ratios and longitude, latitude and altitude **(A–I)**. (Sig. *p* < 0.05).

Linear regression indicated that leaf N% was positively associated with altitude and longitude but negatively associated with latitude (**Figures 2D–F**). Leaf P% exhibited strongly positive correlations with altitude and latitude (**Figures 2H,I**). The leaf C:N ratio was negatively related to altitude and longitude (**Figures 3A,C**). The leaf C:P ratio was negatively correlated with altitude and latitude (**Figures 3E,F**). The leaf N:P ratio related positively to longitude but negatively to latitude (**Figures 3G,H**). The results showed no significant relationship between leaf C% and longitude, latitude and altitude, leaf P% and longitude, C:N and latitude, C:P and longitude and N:P and altitude, respectively (**Figures 2A–C,G**, **3B,D,I**).

The results of a linear mixed effect model analysis indicated that differences in MAT were the major environmental factors determining the variations in leaf N%, P%, C:N, C:P, and N:P ratios for all species, and MAT exhibited strongly negative correlations with leaf N% and P%, but positive correlations with their ratios. pH and MAP affected leaf C% and C:N ratio of all plants from different aspects, respectively. Life forms, species and plot also had significant effects on element contents of all aquatic macrophytes. For submerged plants, MAT was negatively associated with leaf N%, P% but positively associated with C:N and C:P ratios. We also observed that MAP was the most significant environmental factor for floating-leaved plants, which was negatively correlated with leaf N% and N:P ratio, whereas it was positively associated with C:N ratio. Moreover, MAT had a significant positive effect on leaf C% in floating-leaved plants. For emergent plants, MAT displayed strong negative correlations with leaf N%, but it presented strong positive correlations with leaf C%, C:P, and N:P ratios. Furthermore, the effects of Species and Plot on leaf ecological stoichiometry characteristic were reduced in each life forms (**Table 3**). Pearson correlation among environmental factors is shown in **Appendix S3**.

**Table 3:** Summary statistics of linear mixed effect model, which are for the effects of fixed (MAT, mean annual temperature; MAP, mean annual precipitation, pH and Life forms) and random (Species nested in Plot) on leaf C%, N%, P%, and their ratios.

	Variables	d.f.	C% Estimate	N% Estimate	P% Estimate	C:N Estimate	C:P Estimate	N:P Estimate
**All species**								
Fixed	MAT	1	0.093	−0.043^∗∗^	−0.008^∗∗∗^	1.077^∗∗∗^	17.971^∗∗∗^	0.477^∗∗∗^
	MAP	1	0.005	−0.001	−7.085 × 10^−5^	0.028^∗^	0.178	0.002
	pH	1	−0.513^∗^	−0.018	−0.006	0.376	−1.838	−0.084
	Life forms	2	2.487^∗∗∗^	0.027	−0.005	2.517^∗∗∗^	43.151^∗∗∗^	1.269^∗∗∗^
Random	Plot(Species)		5.867^∗^	0.464^∗∗∗^	−1.880 × 10^−6∗^	208.100^∗∗∗^	8399.000	31.223^∗^
**Submerged**								
Fixed	MAT	1	−0.301	−0.011^∗∗∗^	−0.008^∗∗∗^	1.057^∗∗∗^	13.530^∗∗^	0.068
	MAP	1	−0.002	−0.002	−2.534 × 10^−5^	0.030^∗^	0.050	−0.012
	pH	1	−1.107	−0.072	−0.001	0.564	−5.480	−0.761
Random	Plot(Species)		18.040^∗∗^	0.242	3.260 × 10^−4^	45.421^∗∗^	3267.000	3.206
**Floating-leaved**								
Fixed	MAT	1	1.071^∗∗^	−0.010	0.002	0.711	7.207	−0.040
	MAP	1	0.005	−0.011^∗^	−1.610 × 10^−4^	0.097^∗∗∗^	−0.364	−0.089^∗∗^
	pH	1	−0.641	−0.194	−0.073	1.301	42.323	2.282
Random	Plot(Species)		5.647	0.226	0.008	8.056	4456.000	11.572
**Emergent**								
Fixed	MAT	1	0.204^∗∗∗^	−0.131	−0.008^∗∗∗^	1.064^∗^	20.017^∗∗∗^	0.686^∗∗∗^
	MAP	1	0.007^∗^	0.001	−9.020 × 10^−5^	0.022	0.230	0.012
	pH	1	−0.401	0.009	−0.004	0.068	−5.999	−0.006
Random	Plot(Species)		0.435	0.538^∗∗∗^	0.004	296.540^∗∗∗^	12367.000	46.440^∗∗^

*Linear mixed effect model were also made for each life forms. d.f, degrees of freedom. ^∗∗∗^p < 0.001, ^∗∗^p < 0.01, ^∗^p < 0.05.*

## Discussion

### Patterns of Leaf C%, N%, P%, and Their Ratios of Aquatic Macrophytes in the Arid Zone of Northwestern China

Our results showed the mean leaf C% of aquatic plant in the arid zone of northwestern China was 39.95%, approximately identical to the value of aquatic macrophyte in eastern China (36.97%, Xia et al., 2014) and Tibetan Plateau (37.83%, Wang et al., 2015). Our findings with the results of the two abovementioned studies showed that the mean leaf C% of aquatic plant was drastically lower than the value reported by McGroddy et al. (47%, 2004) for forest plants and by He et al. (43.8%, 2006) for Chinese grassland. One possible explanation for this finding is that the low leaf C% results are due to relatively low contents of lignin and cellulose in aquatic plants (Santamaría et al., 2003), because water buoyancy can provide support for aquatic macrophytes, especially for submerged plants.

For leaf N%, our research indicated that mean nitrogen content was 2.12% and closed to 2.02% for terrestrial plants in China (Han et al., 2005). Regarding the leaf P% of aquatic plants in the arid zone of China, the mean value was nearly equal to the mean value for 753 terrestrial plant species in China (0.15%, Han et al., 2005) and 343 aquatic macrophytes species in Spanish shallow lakes (0.13%, Fernández-Aláez et al., 1999). However, compared to two other climate zones in China, the leaf N% and P% of aquatic plant in the arid zone, especially for leaf P%, were substantially below these average values in humid monsoon eastern China (Xia et al., 2014) and the cold area in Tibetan Plateau (Wang et al., 2015). Several previous studies have shown that the mean annual precipitation and temperature can significantly influence leaf nitrogen and phosphorus content (Reich and Oleksyn, 2004; Han et al., 2005; Wang et al., 2015). Thus, the mean values of leaf N% and P% were lower than those from the Tibetan Plateau and eastern China, which may be due to an adaptation strategy of the plants to severe drought. If so, this finding suggests a remarkable climatic characteristic in the arid zone in the long-term evolutionary process. In agreement with other studies (Xia et al., 2014), our study also showed that floating-leaved plants presented significantly higher leaf N% and P% than the other two life forms of plants, which can be explained by that floating-leaved plants can absorb more dissolved N or P from the water and the sediment through their adventitious roots produced from their leaves or stems (Greenway, 1997).

Similarly, our findings also showed that the leaf N:P ratios (16.60) of aquatic plants in the study area were higher than the counterpart values of aquatic macrophytes in eastern China (9.5, Xia et al., 2014) and Tibetan Plateau in China (11.3, Wang et al., 2015). The N:P ratios are always used as one of the important indicators for current restricted nutrient judgement. Several studies showed that plant growth is limited by P contents if the leaf N:P > 16 (Koerselman and Meuleman, 1996). The obtained leaf N:P value in this study suggested that the productivity of aquatic macrophytes in the arid zone of northwestern China may be limited by surrounding P contents.

### Effect of Environmental Factors on the Leaf C%, N%, and P% Stoichiometry

Environmental factors, such as light, temperature, precipitation, and soil nutrients, can significantly affect leaf ecological stoichiometry characteristics (Striebel et al., 2008; Cross et al., 2015; Li et al., 2015; Wang et al., 2015). Temperature plays an important role in ecological stoichiometry characteristics of plants due to the direct effects on plant physiological processes (photosynthesis and respiration) (Weih and Karlsson, 2001; Lacoul and Freedman, 2006). Previous research has found that temperature was the greatest environmental factor affecting leaf stoichiometry of aquatic macrophytes in two other climate zones in China (**Appendix S4**). These findings showed that the leaf N% increased as the temperature decreased in the Tibetan Plateau (Wang et al., 2015), while another study showed the opposite result (Xia et al., 2014). These conflicting results could provide evidence to support TPPH or TBH, respectively. In this study, the leaf N% and P% of all plants recorded increased with decreasing MAT, which supported the TPPH. The higher leaf N% and P% can increase plant metabolic activity to counteract the effect of low temperatures on enzyme activity in plants, in other words, the low temperature promotes the domestication and adaptation of plants (Oleksyn et al., 1998; Tjoelker et al., 1999). We also found that MAT showed positive correlations with the N:P ratios of all plants, which is in agreement with the studies in eastern China (Xia et al., 2014) and the Tibetan Plateau (Wang et al., 2015). A possible explanation is that under lower mean annual temperature conditions, plant with lower leaf N:P ratios can match higher growth rates to resist shorter growth cycles (Güsewell, 2004; Reich and Oleksyn, 2004; Kerkhoff et al., 2006). In addition, MAT had a significant positive effect on leaf C% in floating-leaved plants and emergent plants. This finding may be observed because plants grown at a warmer temperature produced more leaf area and had higher daytime rates of net ecosystem exchange or CO_2_ uptake (Park and Day, 2007). Furthermore, floating-leaved plants and emergent plants need to produce more mechanical tissue of leaves than submerged plants during growth promotion caused by temperature rise.

Precipitation is considered to be another crucial factor that influences the contents of plant leaf elements because drought can affect the cell concentration to enhance the protection of water (Field and Mooney, 1986; Osmond et al., 1987; Seligman and Sinclair, 1995). Several studies showed that the leaf ecological stoichiometry characteristics of terrestrial plants were extremely limited by precipitation (He et al., 2006; He et al., 2008). We found that the C:N ratios of all aquatic macrophytes were positively related to MAP. This result could be due to abundant rainfall increasing the supply of water to reduce the adverse effects of drought, which can lead to a significant addition in plant C:N ratios. Furthermore, our results demonstrate that the leaf C% of emergent plants increased along with MAP, which maybe due to the rapid increase of plant biomass associated with increasing rainfall in dry area (Liu et al., 2012). MAP was found to be the major influencing factor for floating plants, which was negatively correlated with leaf N%. Such allocation pattern is considered to be a functional trade off between roots and leaves (Bloom et al., 1985; Barko et al., 1991), that is, plants have to allocate more internal resources to the organ growth responsible for obtaining external resources that are in lacking supply. This is an adaptability strategy of floating-leaved plants to dynamic nutrient contents caused by water level.

Water physicochemical factors can affect directly or indirectly the elemental distribution of aquatic macrophytes (Kirk, 1983; Cronk and Fennessy, 2001; Lacoul and Freedman, 2006). pH is an important factor influencing ecological stoichiometry characteristics of plants in the arid zone of northwestern China because the water in this region is quite saline and alkaline. We found that there was a significant negative influence of pH on the leaf C% of all aquatic macrophytes. This finding may be observed because under higher pH condition, the decreased concentration of HCO_3_^−^ and dissolved CO_2_ limits the supply of carbon sources in water for aquatic macrophytes (Adams et al., 1978).

Altitude, longitude and latitude are always considered to be geographical factors that affect the large-scale geographic distribution patterns of leaf ecological stoichiometry (Körner, 1989; Reich and Oleksyn, 2004; Han et al., 2005; Li et al., 2015). Our results showed that in the arid zone of northwestern China, the leaf N% of aquatic macrophtes decreased with increasing latitude, whereas the leaf P% increased. One possible explanation for this is the effect of the rate of soils development which depends on rainfall and temperature (Lambers et al., 2008). Moreover, in this study, we found the leaf N% and P% appeared to have strong positive correlations with altitude but strong negative correlations with MAT. This finding is not surprising, since altitude changes in temperature are known to affect leaf N% and P% (Körner, 1989; Soethe et al., 2008; Fisher et al., 2013; Zhao et al., 2014), which may be observed because changes in foliar N% and P% are functional responses to water availability, as altitude is closely linked to potential evapotranspiration and precipitation (Li and Yu, 2009). Thus, the pattern of ecological stoichiometry characteristics of aquatic macrophytes in the study area may be a result of different combinations and interactions of local environmental factors and geographical factors (altitude, longitude, and latitude), which can influence temperature, precipitation, and potential evapotranspiration.

## Conclusion

In this study, we found that the mean values of leaf P% were lower than those in the Tibetan Plateau and eastern China, and P may be the limiting factor of the macrophyte communities in the arid zone of northwestern China. Our results supported the temperature-plant physiology hypothesis owing to significant, negative relationships between leaf N% and P% of macrophytes and mean annual temperature. Our results also showed strong relationships between leaf N% and N:P ratios and longitude, leaf N%, P%, and N:P ratios and latitude, and leaf N% and P% and altitude, respectively. In addition, the results showed that pH can significantly influence leaf C%. The different patterns of leaf stoichiometry between the arid zone of northwestern China and eastern China indicated that there were different physiological and ecological adaptability of macrophytes to environmental gradients in different climatic zones. Compared to prior studies, our study suggests the determinants that influence leaf carbon, nitrogen, and phosphorus stoichiometry of aquatic macrophytes differed among different climatic zones. Thus, further research is warranted to investigate leaf stoichiometry patterns and the primary influencing factors on larger spatial scales. Also, we only studied the effect of three environmental factors (mean annual temperature, mean annual precipitation, pH) on the leaf C, N, and P stoichiometry. It will be of interest in the future to analyse in detail the effects of other factors, such as soil physicochemical characteristics and water physicochemical characteristics.

## Author Contributions

ZL and JZ designed the research. XG, ZX, WL, YT, and YL collected and analyzed samples. ZL, JZ, XG, ZW, and CD analyzed and discussed the data. XG and ZL wrote the manuscript.

## Conflict of Interest Statement

The authors declare that the research was conducted in the absence of any commercial or financial relationships that could be construed as a potential conflict of interest.
