# Supplementary material for: Spatial Patterns of Leaf Carbon, Nitrogen, and Phosphorus Stoichiometry of Aquatic Macrophytes in the Arid Zone of Northwestern China
Source: Front Plant Sci. 2018 Sep 21;9:1398. doi: 10.3389/fpls.2018.01398 (PMC6160588; doi:10.3389/fpls.2018.01398)
Supplement: Supplementary file 1 [file Table_1.DOC]

**Supporting informations**

**Appendix S1** Raw data of species list, life form, carbon (C%), nitrgen (N%) and phosphorus concentration (P%) for all observations examined. Multi-annual mean temperature (MAT, ^o^C), multi-annual mean precipitation (MAP, mm) and pH were also provided.

| **Species** | **Life form** | **C%** | **N%** | **P%** | **MAT** | **MAP** | **pH** |
| --- | --- | --- | --- | --- | --- | --- | --- |
| *Acorus calamus* | Emergent | 42.29 | 1.76 | 0.15 | 8.39 | 134.24 | 7.60 |
| *Alisma canaliculatum* | Emergent | 37.38 | 3.29 | 0.28 | 1.56 | 231.08 | 9.70 |
| *Alisma canaliculatum* | Emergent | 40.21 | 4.68 | 0.28 | 1.56 | 231.08 | 9.70 |
| *Alisma canaliculatum* | Emergent | 38.68 | 3.11 | 0.22 | 4.95 | 204.75 | 8.60 |
| *Alisma canaliculatum* | Emergent | 35.94 | 2.63 | 0.13 | 3.39 | 194.21 | 8.60 |
| *Alisma canaliculatum* | Emergent | 39.62 | 2.71 | 0.35 | 4.85 | 208.27 | 8.20 |
| *Alisma canaliculatum* | Emergent | 40.35 | 1.38 | 0.24 | 5.32 | 187.90 | 7.90 |
| *Alisma canaliculatum* | Emergent | 39.23 | 2.26 | 0.18 | 5.31 | 198.90 | 7.90 |
| *Alisma gramineum* | Emergent | 44.58 | 1.59 | 0.17 | 5.34 | 198.63 | 8.90 |
| *Alisma gramineum* | Emergent | 33.47 | 2.57 | 0.17 | 5.34 | 198.63 | 8.90 |
| *Alisma nanum* | Emergent | 43.21 | 1.15 | 0.16 | 5.34 | 198.63 | 8.90 |
| *Alisma nanum* | Emergent | 41.62 | 1.85 | 0.15 | 5.31 | 198.73 | 8.50 |
| *Alisma nanum* | Emergent | 43.62 | 3.36 | 0.19 | 8.51 | 96.62 | 8.00 |
| *Alisma nanum* | Emergent | 41.97 | 2.45 | 0.31 | 2.74 | 191.10 | 7.10 |
| *Alisma orientale* | Emergent | 41.81 | 4.07 | 0.34 | 5.31 | 198.73 | 8.50 |
| *Alisma orientale* | Emergent | 42.66 | 2.91 | 0.19 | 4.54 | 213.13 | 8.10 |
| *Alisma orientale* | Emergent | 40.79 | 3.31 | 0.24 | 7.88 | 214.74 | 7.90 |
| *Alisma orientale* | Emergent | 42.76 | 3.86 | 0.16 | 4.14 | 363.56 | 7.90 |
| *Alisma orientale* | Emergent | 41.82 | 3.23 | 0.16 | 8.39 | 134.24 | 7.60 |
| *Alisma plantago-aquatica* | Emergent | 41.73 | 2.99 | 0.09 | 3.21 | 135.96 | 9.90 |
| *Alisma plantago-aquatica* | Emergent | 42.68 | 3.13 | 0.19 | 4.79 | 172.44 | 8.90 |
| *Alisma plantago-aquatica* | Emergent | 39.06 | 3.09 | 0.21 | 1.47 | 229.85 | 8.60 |
| *Alisma plantago-aquatica* | Emergent | 37.53 | 1.93 | 0.14 | 5.22 | 204.95 | 8.30 |
| *Alisma plantago-aquatica* | Emergent | 41.38 | 2.46 | 0.14 | 2.82 | 138.57 | 8.20 |
| *Alisma plantago-aquatica* | Emergent | 41.85 | 3.17 | 0.18 | 3.11 | 213.91 | 7.90 |
| *Alisma plantago-aquatica* | Emergent | 40.91 | 2.19 | 0.10 | 3.11 | 213.91 | 7.90 |
| *Alisma plantago-aquatica* | Emergent | 40.18 | 1.67 | 0.20 | 5.16 | 203.43 | 7.70 |
| *Alisma plantago-aquatica* | Emergent | 40.83 | 3.80 | 0.21 | 2.83 | 138.42 | 7.70 |
| *Alisma plantago-aquatica* | Emergent | 41.67 | 3.32 | 0.19 | 2.05 | 199.44 | 7.30 |
| *Alisma plantago-aquatica* | Emergent | 41.63 | 3.98 | 0.25 | 2.74 | 191.10 | 7.10 |
| *Batrachium bungei* | Submerged | 37.29 | 3.71 | 0.35 | -1.31 | 214.46 | 9.90 |
| *Batrachium bungei* | Submerged | 43.59 | 2.75 | 0.15 | 1.56 | 231.08 | 9.70 |
| *Batrachium bungei* | Submerged | 37.20 | 3.02 | 0.20 | -4.02 | 237.59 | 9.50 |
| *Batrachium bungei* | Submerged | 33.67 | 1.07 | 0.09 | 1.47 | 229.85 | 8.60 |
| *Batrachium bungei* | Submerged | 39.78 | 2.88 | 0.11 | 3.41 | 143.42 | 8.50 |
| *Batrachium bungei* | Submerged | 35.44 | 0.88 | 0.19 | 5.32 | 187.90 | 7.90 |
| *Batrachium bungei* | Submerged | 32.34 | 2.39 | 0.12 | 4.14 | 363.56 | 7.90 |
| *Batrachium bungei* | Submerged | 32.99 | 2.47 | 0.11 | 2.64 | 226.09 | 7.40 |
| *Batrachium bungei* | Submerged | 39.79 | 2.71 | 0.25 | 2.05 | 199.44 | 7.30 |
| *Batrachium bungei* | Submerged | 40.35 | 3.47 | 0.14 | -0.36 | 265.34 | 8.80 |
| *Butomus umbellatus* | Emergent | 42.38 | 0.66 | 0.11 | 5.34 | 198.63 | 8.90 |
| *Butomus umbellatus* | Emergent | 38.30 | 2.45 | 0.27 | 4.79 | 172.44 | 8.90 |
| *Butomus umbellatus* | Emergent | 45.08 | 1.21 | 0.16 | 5.22 | 204.95 | 8.30 |
| *Butomus umbellatus* | Emergent | 37.05 | 1.94 | 0.25 | 4.85 | 208.27 | 8.20 |
| *Butomus umbellatus* | Emergent | 38.01 | 1.34 | 0.15 | 4.54 | 213.13 | 8.10 |
| *Butomus umbellatus* | Emergent | 40.69 | 2.22 | 0.08 | 5.32 | 187.90 | 7.90 |
| *Callitriche palustris* | Submerged | 44.03 | 3.05 | 0.40 | -0.36 | 265.34 | 8.80 |
| *Callitriche stagnalis* | Submerged | 38.93 | 2.22 | 0.27 | 5.31 | 198.73 | 8.50 |
| *Callitriche stagnalis* | Submerged | 38.94 | 2.03 | 0.19 | 2.05 | 199.44 | 7.30 |
| *Carex amgunensis* | Emergent | 37.39 | 2.16 | 0.14 | 4.79 | 172.44 | 8.90 |
| *Carex amgunensis* | Emergent | 42.04 | 1.41 | 0.20 | -0.36 | 265.34 | 8.80 |
| *Carex amgunensis* | Emergent | 44.18 | 1.27 | 0.37 | 5.31 | 198.73 | 8.50 |
| *Carex amgunensis* | Emergent | 40.16 | 3.02 | 0.14 | 4.92 | 164.73 | 8.40 |
| *Carex amgunensis* | Emergent | 41.72 | 3.19 | 0.21 | 3.11 | 213.91 | 7.90 |
| *Carex amgunensis* | Emergent | 43.65 | 2.93 | 0.22 | 7.72 | 131.23 | 7.80 |
| *Carex amgunensis* | Emergent | 44.56 | 1.67 | 0.21 | 2.05 | 199.44 | 7.30 |
| *Carex amgunensis* | Emergent | 44.89 | 0.80 | 0.14 | 2.74 | 191.10 | 7.10 |
| *Ceratophyllum demersum* | Submerged | 38.65 | 1.79 | 0.11 | 4.54 | 213.13 | 8.10 |
| *Ceratophyllum demersum* | Submerged | 34.29 | 2.18 | 0.34 | 5.32 | 187.90 | 7.90 |
| *Ceratophyllum demersum* | Submerged | 40.43 | 2.11 | 0.10 | 8.77 | 91.13 | 7.90 |
| *Ceratophyllum demersum* | Submerged | 40.03 | 2.12 | 0.11 | 8.73 | 91.34 | 7.80 |
| *Ceratophyllum demersum* | Submerged | 40.91 | 2.89 | 0.15 | 8.79 | 92.27 | 7.80 |
| *Ceratophyllum demersum* | Submerged | 40.37 | 2.53 | 0.12 | 8.70 | 91.79 | 7.80 |
| *Ceratophyllum demersum* | Submerged | 41.56 | 2.11 | 0.10 | 8.81 | 91.39 | 7.80 |
| *Ceratophyllum demersum* | Submerged | 41.39 | 1.79 | 0.07 | 8.79 | 92.27 | 7.80 |
| *Ceratophyllum demersum* | Submerged | 37.67 | 2.84 | 0.21 | 8.78 | 92.73 | 7.70 |
| *Ceratophyllum demersum* | Submerged | 38.78 | 2.24 | 0.10 | 8.74 | 91.48 | 7.70 |
| *Chara* | Submerged | 19.67 | 0.71 | 0.05 | 2.32 | 220.94 | 9.60 |
| *Chara* | Submerged | 26.87 | 1.40 | 0.11 | 8.44 | 97.84 | 9.10 |
| *Chara* | Submerged | 23.14 | 1.16 | 0.09 | 3.81 | 185.63 | 8.90 |
| *Chara* | Submerged | 19.83 | 0.60 | 0.07 | 1.47 | 229.85 | 8.60 |
| *Chara* | Submerged | 24.11 | 0.87 | 0.07 | 8.64 | 93.61 | 8.30 |
| *Chara* | Submerged | 23.02 | 0.71 | 0.04 | 8.59 | 94.47 | 8.30 |
| *Chara* | Submerged | 22.53 | 0.77 | 0.10 | 4.85 | 208.27 | 8.20 |
| *Chara* | Submerged | 25.34 | 1.07 | 0.12 | 5.20 | 195.20 | 8.20 |
| *Chara* | Submerged | 20.88 | 0.57 | 0.06 | 4.54 | 213.13 | 8.10 |
| *Chara* | Submerged | 20.16 | 0.89 | 0.07 | 4.96 | 198.32 | 8.00 |
| *Chara* | Submerged | 19.69 | 1.02 | 0.08 | 9.13 | 50.67 | 8.00 |
| *Chara* | Submerged | 20.27 | 0.98 | 0.08 | 13.86 | 32.71 | 8.00 |
| *Chara* | Submerged | 22.40 | 0.72 | 0.05 | 9.37 | 87.35 | 8.00 |
| *Chara* | Submerged | 41.48 | 3.04 | 0.13 | 9.96 | 76.25 | 8.00 |
| *Chara* | Submerged | 22.97 | 0.56 | 0.06 | 5.31 | 198.90 | 7.90 |
| *Chara* | Submerged | 29.18 | 1.49 | 0.08 | 9.46 | 140.94 | 7.90 |
| *Chara* | Submerged | 29.34 | 1.33 | 0.16 | 8.81 | 91.62 | 7.80 |
| *Chara* | Submerged | 21.29 | 1.41 | 0.11 | 9.60 | 7.90 | 7.80 |
| *Chara* | Submerged | 29.50 | 1.30 | 0.09 | 8.81 | 91.13 | 7.80 |
| *Chara* | Submerged | 36.16 | 1.74 | 0.08 | 8.81 | 91.39 | 7.80 |
| *Chara* | Submerged | 25.14 | 1.49 | 0.09 | 7.82 | 126.63 | 7.70 |
| *Chara* | Submerged | 38.87 | 2.76 | 0.16 | 3.67 | 173.47 | 7.70 |
| *Chara* | Submerged | 36.73 | 2.53 | 0.20 | 2.05 | 199.44 | 7.30 |
| *Chara* | Submerged | 36.34 | 2.05 | 0.06 | 8.79 | 91.11 | 7.80 |
| *Cyperus difformis* | Emergent | 41.80 | 0.78 | 0.13 | 5.22 | 204.95 | 8.30 |
| *Cyperus difformis* | Emergent | 42.89 | 1.03 | 0.13 | 13.86 | 32.71 | 8.00 |
| *Cyperus difformis* | Emergent | 42.39 | 0.95 | 0.13 | 9.46 | 140.94 | 7.90 |
| *Cyperus difformis* | Emergent | 35.33 | 0.67 | 0.06 | 12.45 | 55.96 | 7.90 |
| *Cyperus difformis* | Emergent | 30.04 | 0.58 | 0.03 | 7.80 | 127.22 | 7.70 |
| *Cyperus iria* | Emergent | 41.92 | 1.53 | 0.16 | 5.22 | 204.95 | 8.30 |
| *Cyperus iria* | Emergent | 43.43 | 1.38 | 0.10 | 7.82 | 126.63 | 7.70 |
| *Echinochloa caudata* | Emergent | 42.68 | 2.67 | 0.14 | 7.01 | 118.71 | 7.80 |
| *Echinochloa colonum* | Emergent | 44.13 | 1.60 | 0.16 | 2.82 | 138.57 | 8.20 |
| *Echinochloa colonum* | Emergent | 41.84 | 1.97 | 0.20 | 2.31 | 141.97 | 7.40 |
| *Echinochloa phyllopogon* | Emergent | 42.31 | 1.17 | 0.22 | 8.71 | 91.59 | 8.20 |
| *Echinochloa phyllopogon* | Emergent | 43.06 | 1.56 | 0.14 | 8.62 | 93.61 | 8.20 |
| *Echinochloa phyllopogon* | Emergent | 43.29 | 0.87 | 0.06 | 12.45 | 55.96 | 7.90 |
| *Echinochloa phyllopogon* | Emergent | 42.03 | 1.46 | 0.10 | 9.46 | 140.94 | 7.90 |
| *Echinochloa phyllopogon* | Emergent | 42.95 | 5.04 | 0.11 | 4.14 | 363.56 | 7.90 |
| *Epilobium hirsutum* | Emergent | 42.47 | 1.35 | 0.14 | 4.85 | 208.27 | 8.20 |
| *Epilobium hirsutum* | Emergent | 43.77 | 4.27 | 0.20 | 12.45 | 55.96 | 7.90 |
| *Epilobium hirsutum* | Emergent | 44.37 | 0.92 | 0.05 | 5.16 | 203.43 | 7.70 |
| *Epilobium palustre* | Emergent | 43.92 | 5.45 | 0.15 | 7.88 | 214.74 | 7.90 |
| *Equisetum hyemale* | Emergent | 36.18 | 1.70 | 0.03 | 4.79 | 172.44 | 8.90 |
| *Equisetum hyemale* | Emergent | 38.29 | 1.88 | 0.03 | 5.31 | 198.73 | 8.50 |
| *Equisetum hyemale* | Emergent | 38.87 | 2.09 | 0.14 | 2.05 | 199.44 | 7.30 |
| *Equisetum hyemale* | Emergent | 36.07 | 1.77 | 0.10 | 2.74 | 191.10 | 7.10 |
| *Halerpestes cymbalaria* | Emergent | 42.61 | 1.75 | 0.14 | -3.36 | 232.42 | 10.00 |
| *Halerpestes cymbalaria* | Emergent | 42.11 | 3.05 | 0.22 | -3.36 | 232.42 | 10.00 |
| *Halerpestes cymbalaria* | Emergent | 42.39 | 3.27 | 0.13 | -1.31 | 214.46 | 9.90 |
| *Halerpestes cymbalaria* | Emergent | 40.97 | 4.00 | 0.25 | 1.56 | 231.08 | 9.70 |
| *Halerpestes cymbalaria* | Emergent | 40.84 | 2.45 | 0.21 | 2.32 | 220.94 | 9.60 |
| *Halerpestes cymbalaria* | Emergent | 40.51 | 2.58 | 0.19 | 1.99 | 231.26 | 9.50 |
| *Halerpestes cymbalaria* | Emergent | 44.91 | 3.86 | 0.22 | -4.02 | 237.59 | 9.50 |
| *Halerpestes cymbalaria* | Emergent | 42.72 | 3.78 | 0.36 | -0.36 | 265.34 | 8.80 |
| *Halerpestes cymbalaria* | Emergent | 39.99 | 1.59 | 0.20 | 1.47 | 229.85 | 8.60 |
| *Halerpestes cymbalaria* | Emergent | 42.69 | 2.74 | 0.19 | -15.12 | 504.81 | 8.50 |
| *Halerpestes cymbalaria* | Emergent | 44.85 | 1.52 | 0.22 | 5.22 | 204.95 | 8.30 |
| *Halerpestes cymbalaria* | Emergent | 42.37 | 1.91 | 0.14 | 8.51 | 96.62 | 8.00 |
| *Halerpestes cymbalaria* | Emergent | 40.49 | 3.41 | 0.22 | 4.96 | 198.32 | 8.00 |
| *Halerpestes cymbalaria* | Emergent | 39.66 | 1.97 | 0.13 | 3.11 | 213.91 | 7.90 |
| *Halerpestes cymbalaria* | Emergent | 39.65 | 3.18 | 0.06 | 9.46 | 140.94 | 7.90 |
| *Halerpestes cymbalaria* | Emergent | 41.25 | 2.48 | 0.28 | 3.67 | 173.47 | 7.70 |
| *Halerpestes tricuspis* | Emergent | 42.07 | 1.59 | 0.12 | 3.21 | 135.96 | 9.90 |
| *Halerpestes tricuspis* | Emergent | 37.37 | 2.72 | 0.15 | 10.19 | -22.47 | 8.70 |
| *Halerpestes tricuspis* | Emergent | 39.49 | 1.35 | 0.07 | 1.47 | 229.85 | 8.60 |
| *Halerpestes tricuspis* | Emergent | 37.51 | 3.16 | 0.19 | 5.20 | 195.20 | 8.20 |
| *Halerpestes tricuspis* | Emergent | 44.04 | 2.69 | 0.19 | 7.82 | 126.63 | 7.70 |
| *Halerpestes tricuspis* | Emergent | 40.27 | 1.64 | 0.23 | 2.05 | 199.44 | 7.30 |
| *Heleocharis dulcis* | Emergent | 40.52 | 3.18 | 0.32 | -0.36 | 265.34 | 8.80 |
| *Heleocharis fennica* | Emergent | 41.73 | 2.19 | 0.15 | 4.79 | 172.44 | 8.90 |
| *Heleocharis fennica* | Emergent | 42.00 | 1.08 | 0.09 | 9.13 | 50.67 | 8.00 |
| *Heleocharis fennica* | Emergent | 42.90 | 1.27 | 0.26 | 2.05 | 199.44 | 7.30 |
| *Heleocharis pellucida* | Emergent | 42.53 | 1.14 | 0.11 | 7.88 | 214.74 | 7.90 |
| *Heleocharis pellucida* | Emergent | 40.35 | 3.56 | 0.14 | 4.14 | 363.56 | 7.90 |
| *Heleocharis pellucida* | Emergent | 41.15 | 2.17 | 0.22 | 1.56 | 231.08 | 9.70 |
| *Heleocharis pellucida* | Emergent | 40.86 | 0.72 | 0.06 | 2.32 | 220.94 | 9.60 |
| *Heleocharis pellucida* | Emergent | 41.48 | 2.17 | 0.17 | 1.99 | 231.26 | 9.50 |
| *Heleocharis pellucida* | Emergent | 41.69 | 2.04 | 0.26 | -0.36 | 265.34 | 8.80 |
| *Heleocharis pellucida* | Emergent | 37.32 | 3.80 | 0.09 | 4.44 | 134.98 | 8.60 |
| *Heleocharis pellucida* | Emergent | 41.66 | 2.92 | 0.16 | 5.31 | 198.73 | 8.50 |
| *Heleocharis pellucida* | Emergent | 39.58 | 3.69 | 0.17 | 3.41 | 143.42 | 8.50 |
| *Heleocharis pellucida* | Emergent | 41.35 | 0.81 | 0.15 | 5.20 | 195.20 | 8.20 |
| *Heleocharis pellucida* | Emergent | 41.07 | 1.19 | 0.13 | 4.85 | 208.27 | 8.20 |
| *Heleocharis pellucida* | Emergent | 39.18 | 2.65 | 0.16 | 4.02 | 138.27 | 8.20 |
| *Heleocharis pellucida* | Emergent | 40.46 | 1.01 | 0.11 | 13.86 | 32.71 | 8.00 |
| *Heleocharis pellucida* | Emergent | 36.23 | 2.65 | 0.22 | 2.95 | 147.12 | 8.00 |
| *Heleocharis pellucida* | Emergent | 40.19 | 1.19 | 0.07 | 4.96 | 198.32 | 8.00 |
| *Heleocharis pellucida* | Emergent | 40.92 | 2.60 | 0.07 | 8.98 | 137.45 | 8.00 |
| *Heleocharis pellucida* | Emergent | 42.08 | 0.93 | 0.19 | 5.32 | 187.90 | 7.90 |
| *Heleocharis pellucida* | Emergent | 41.59 | 0.83 | 0.10 | 9.46 | 140.94 | 7.90 |
| *Heleocharis pellucida* | Emergent | 37.72 | 1.51 | 0.15 | 3.11 | 213.91 | 7.90 |
| *Heleocharis pellucida* | Emergent | 41.82 | 2.00 | 0.10 | 5.31 | 198.90 | 7.90 |
| *Heleocharis pellucida* | Emergent | 42.29 | 2.68 | 0.22 | 5.27 | 199.71 | 7.80 |
| *Heleocharis pellucida* | Emergent | 43.00 | 3.03 | 1.15 | 3.67 | 173.47 | 7.70 |
| *Heleocharis pellucida* | Emergent | 41.65 | 2.64 | 0.21 | 2.83 | 138.42 | 7.70 |
| *Heleocharis pellucida* | Emergent | 34.15 | 1.52 | 0.09 | 8.39 | 134.24 | 7.60 |
| *Heleocharis pellucida* | Emergent | 39.34 | 0.89 | 0.05 | 5.52 | 189.48 | 7.60 |
| *Heleocharis plantagineiformis* | Emergent | 25.27 | 0.54 | 0.05 | 2.82 | 138.57 | 8.20 |
| *Heleocharis plantagineiformis* | Emergent | 40.38 | 2.25 | 0.24 | 9.13 | 50.67 | 8.00 |
| *Heleocharis plantagineiformis* | Emergent | 40.88 | 1.92 | 0.10 | 7.72 | 131.23 | 7.80 |
| *Heleocharis yokoscensis* | Emergent | 40.33 | 2.67 | 0.20 | -0.36 | 265.34 | 8.80 |
| *Heleocharis yokoscensis* | Emergent | 39.88 | 2.97 | 0.32 | 2.05 | 199.44 | 7.30 |
| *Hippuris vulgaris* | Emergent | 38.97 | 2.21 | 0.21 | 3.21 | 135.96 | 9.90 |
| *Hippuris vulgaris* | Emergent | 39.33 | 2.28 | 0.18 | 2.32 | 220.94 | 9.60 |
| *Hippuris vulgaris* | Emergent | 38.64 | 1.85 | 0.09 | 3.19 | 136.34 | 9.10 |
| *Hippuris vulgaris* | Emergent | 38.97 | 1.29 | 0.15 | 2.82 | 138.57 | 8.20 |
| *Hippuris vulgaris* | Emergent | 44.02 | 2.17 | 0.06 | 5.20 | 195.20 | 8.20 |
| *Hippuris vulgaris* | Emergent | 39.63 | 4.19 | 0.19 | 9.46 | 140.94 | 7.90 |
| *Hippuris vulgaris* | Emergent | 43.44 | 4.55 | 0.13 | 4.14 | 363.56 | 7.90 |
| *Hippuris vulgaris* | Emergent | 29.28 | 1.99 | 0.15 | 2.83 | 138.42 | 7.70 |
| *Hippuris vulgaris* | Emergent | 40.09 | 1.68 | 0.12 | 2.74 | 191.10 | 7.10 |
| *Juncellus pannonicus* | Emergent | 42.33 | 0.57 | 0.14 | 5.34 | 198.63 | 8.90 |
| *Juncellus pannonicus* | Emergent | 40.84 | 1.15 | 0.08 | 7.80 | 127.22 | 7.70 |
| *Juncellus serotinus* | Emergent | 44.70 | 0.50 | 0.11 | 5.34 | 198.63 | 8.90 |
| *Juncellus serotinus* | Emergent | 35.39 | 1.40 | 0.25 | 4.85 | 208.27 | 8.20 |
| *Juncellus serotinus* | Emergent | 41.10 | 1.19 | 0.14 | 8.71 | 91.59 | 8.20 |
| *Juncellus serotinus* | Emergent | 42.27 | 0.27 | 0.06 | 8.51 | 96.80 | 8.10 |
| *Juncellus serotinus* | Emergent | 39.85 | 0.82 | 0.14 | 4.54 | 213.13 | 8.10 |
| *Juncellus serotinus* | Emergent | 42.44 | 0.40 | 0.04 | 13.86 | 32.71 | 8.00 |
| *Juncellus serotinus* | Emergent | 40.44 | 0.66 | 0.13 | 9.48 | 141.17 | 7.90 |
| *Juncellus serotinus* | Emergent | 42.67 | 1.73 | 0.14 | 12.45 | 55.96 | 7.90 |
| *Juncus alatus* | Emergent | 43.30 | 1.58 | 0.14 | 7.88 | 214.74 | 7.90 |
| *Juncus alatus* | Emergent | 43.48 | 0.61 | 0.15 | 5.34 | 198.63 | 8.90 |
| *Juncus alatus* | Emergent | 43.29 | 1.10 | 0.07 | 7.82 | 126.63 | 7.70 |
| *Juncus alatus* | Emergent | 42.79 | 0.68 | 0.06 | 8.39 | 134.24 | 7.60 |
| *Juncus alatus* | Emergent | 45.43 | 1.89 | 0.16 | 2.74 | 191.10 | 7.10 |
| *Juncus effusus* | Emergent | 43.66 | 2.06 | 0.16 | 5.31 | 198.73 | 8.50 |
| *Juncus effusus* | Emergent | 40.89 | 0.85 | 0.11 | 4.85 | 208.27 | 8.20 |
| *Juncus effusus* | Emergent | 42.71 | 1.65 | 0.13 | 13.86 | 32.71 | 8.00 |
| *Juncus effusus* | Emergent | 43.12 | 1.30 | 0.10 | 4.96 | 198.32 | 8.00 |
| *Juncus effusus* | Emergent | 44.04 | 1.09 | 0.05 | 9.46 | 140.94 | 7.90 |
| *Juncus effusus* | Emergent | 42.61 | 0.40 | 0.06 | 5.27 | 199.71 | 7.80 |
| *Juncus effusus* | Emergent | 44.32 | 0.97 | 0.12 | 5.16 | 203.43 | 7.70 |
| *Juncus effusus* | Emergent | 44.34 | 1.17 | 0.10 | 3.67 | 173.47 | 7.70 |
| *Juncus effusus* | Emergent | 44.51 | 0.78 | 0.04 | 5.52 | 189.48 | 7.60 |
| *Lemna minor* | Floating-leaved | 35.80 | 2.89 | 0.12 | 3.21 | 135.96 | 9.90 |
| *Lemna minor* | Floating-leaved | 40.34 | 1.19 | 0.17 | 4.85 | 208.27 | 8.20 |
| *Lemna minor* | Floating-leaved | 32.28 | 2.15 | 0.13 | 2.82 | 138.57 | 8.20 |
| *Lemna minor* | Floating-leaved | 35.00 | 2.10 | 0.16 | 4.54 | 213.13 | 8.10 |
| *Lemna minor* | Floating-leaved | 37.98 | 1.61 | 0.21 | 7.88 | 214.74 | 7.90 |
| *Lemna minor* | Floating-leaved | 35.46 | 1.72 | 0.23 | 5.16 | 203.43 | 7.70 |
| *Lemna minor* | Floating-leaved | 38.96 | 4.77 | 0.24 | 7.80 | 127.22 | 7.70 |
| *Lemna minor* | Floating-leaved | 28.45 | 1.34 | 0.29 | 2.05 | 199.44 | 7.30 |
| *Lemna minor* | Floating-leaved | 42.78 | 2.61 | 0.20 | 2.74 | 191.10 | 7.10 |
| *Limosella aquatica* | Emergent | 41.54 | 3.86 | 0.56 | -0.36 | 265.34 | 8.80 |
| *Limosella aquatica* | Emergent | 38.14 | 3.49 | 0.29 | 2.31 | 141.97 | 7.40 |
| *Limosella aquatica* | Emergent | 42.03 | 3.10 | 0.26 | 2.74 | 191.10 | 7.10 |
| *Myriophyllum propinquum* | Submerged | 35.09 | 2.00 | 0.11 | 3.19 | 136.34 | 9.10 |
| *Myriophyllum propinquum* | Submerged | 39.40 | 3.58 | 0.27 | -0.36 | 265.34 | 8.80 |
| *Myriophyllum propinquum* | Submerged | 38.43 | 2.39 | 0.14 | 2.83 | 138.42 | 7.70 |
| *Myriophyllum spicatum* | Submerged | 37.09 | 2.81 | 0.18 | 3.21 | 135.96 | 9.90 |
| *Myriophyllum spicatum* | Submerged | 35.69 | 1.87 | 0.08 | 2.32 | 220.94 | 9.60 |
| *Myriophyllum spicatum* | Submerged | 25.94 | 0.74 | 0.10 | 1.99 | 231.26 | 9.50 |
| *Myriophyllum spicatum* | Submerged | 27.96 | 1.54 | 0.08 | 3.19 | 136.34 | 9.10 |
| *Myriophyllum spicatum* | Submerged | 38.89 | 1.38 | 0.07 | 5.34 | 198.63 | 8.90 |
| *Myriophyllum spicatum* | Submerged | 35.19 | 1.50 | 0.16 | 6.00 | 180.64 | 8.50 |
| *Myriophyllum spicatum* | Submerged | 37.75 | 2.10 | 0.16 | 8.56 | 94.40 | 8.40 |
| *Myriophyllum spicatum* | Submerged | 29.58 | 1.47 | 0.10 | 5.22 | 204.95 | 8.30 |
| *Myriophyllum spicatum* | Submerged | 39.20 | 1.63 | 0.11 | 8.64 | 93.34 | 8.30 |
| *Myriophyllum spicatum* | Submerged | 37.70 | 1.10 | 0.07 | 9.23 | 140.67 | 8.30 |
| *Myriophyllum spicatum* | Submerged | 36.57 | 1.59 | 0.05 | 8.59 | 94.47 | 8.30 |
| *Myriophyllum spicatum* | Submerged | 40.82 | 2.97 | 0.22 | 8.71 | 91.59 | 8.20 |
| *Myriophyllum spicatum* | Submerged | 38.64 | 2.71 | 0.19 | 8.62 | 93.61 | 8.20 |
| *Myriophyllum spicatum* | Submerged | 38.76 | 3.54 | 0.23 | 8.51 | 96.80 | 8.10 |
| *Myriophyllum spicatum* | Submerged | 40.29 | 2.61 | 0.11 | 9.37 | 87.35 | 8.00 |
| *Myriophyllum spicatum* | Submerged | 37.15 | 2.20 | 0.11 | 7.88 | 214.74 | 7.90 |
| *Myriophyllum spicatum* | Submerged | 40.31 | 0.80 | 0.10 | 8.81 | 91.62 | 7.80 |
| *Myriophyllum spicatum* | Submerged | 34.24 | 1.48 | 0.08 | 8.70 | 91.79 | 7.80 |
| *Myriophyllum spicatum* | Submerged | 28.29 | 1.77 | 0.12 | 7.82 | 126.63 | 7.70 |
| *Myriophyllum verticillatum* | Submerged | 28.72 | 1.70 | 0.14 | 4.93 | 187.99 | 8.30 |
| *Myriophyllum verticillatum* | Submerged | 38.36 | 1.45 | 0.10 | 8.81 | 90.99 | 7.70 |
| *Myriophyllum verticillatum* | Submerged | 32.22 | 2.73 | 0.16 | 2.32 | 220.94 | 9.60 |
| *Myriophyllum verticillatum* | Submerged | 39.19 | 2.24 | 0.16 | 5.34 | 198.63 | 8.90 |
| *Myriophyllum verticillatum* | Submerged | 35.87 | 2.61 | 0.18 | 4.85 | 208.27 | 8.20 |
| *Myriophyllum verticillatum* | Submerged | 37.31 | 1.62 | 0.12 | 5.27 | 199.71 | 7.80 |
| *Myriophyllum verticillatum* | Submerged | 42.28 | 0.72 | 0.05 | 8.81 | 91.39 | 7.80 |
| *Myriophyllum verticillatum* | Submerged | 39.44 | 1.94 | 0.12 | 5.16 | 203.43 | 7.70 |
| *Najas graminea* | Submerged | 38.17 | 1.57 | 0.13 | 5.34 | 198.63 | 8.90 |
| *Najas marina* | Submerged | 29.84 | 1.72 | 0.17 | 6.01 | 179.95 | 9.20 |
| *Najas marina* | Submerged | 33.30 | 1.56 | 0.11 | 5.34 | 198.63 | 8.90 |
| *Najas marina* | Submerged | 40.27 | 0.67 | 0.25 | 6.00 | 180.64 | 8.50 |
| *Najas marina* | Submerged | 34.00 | 1.51 | 0.11 | 8.56 | 94.40 | 8.40 |
| *Najas marina* | Submerged | 33.12 | 2.23 | 0.12 | 8.59 | 94.47 | 8.30 |
| *Najas marina* | Submerged | 36.84 | 2.00 | 0.16 | 8.51 | 96.62 | 8.00 |
| *Najas marina* | Submerged | 29.58 | 0.92 | 0.09 | 9.46 | 140.94 | 7.90 |
| *Najas marina* | Submerged | 40.00 | 1.09 | 0.11 | 4.54 | 213.13 | 8.10 |
| *Nymphaea tetragona* | Floating-leaved | 44.74 | 4.02 | 0.17 | 8.77 | 91.13 | 7.90 |
| *Nymphaea tetragona* | Floating-leaved | 44.19 | 3.67 | 0.21 | 8.70 | 91.79 | 7.80 |
| *Nymphaea tetragona* | Floating-leaved | 42.43 | 2.94 | 0.12 | 8.79 | 91.11 | 7.80 |
| *Nymphaea tetragona* | Floating-leaved | 42.45 | 2.67 | 0.08 | 8.81 | 91.13 | 7.80 |
| *Nymphaea tetragona* | Floating-leaved | 43.31 | 3.40 | 0.94 | 8.74 | 91.48 | 7.70 |
| *Nymphaea tetragona* | Floating-leaved | 43.30 | 2.79 | 0.15 | 8.81 | 90.99 | 7.70 |
| *Nymphoides peltatum* | Floating-leaved | 41.73 | 2.51 | 0.12 | 8.74 | 91.48 | 7.70 |
| *Oenanthe javanica* | Emergent | 42.27 | 1.71 | 0.22 | -0.36 | 265.34 | 8.80 |
| *Oenanthe javanica* | Emergent | 39.99 | 3.76 | 0.11 | 7.88 | 214.74 | 7.90 |
| *Oenanthe javanica* | Emergent | 43.84 | 3.10 | 0.12 | 2.05 | 199.44 | 7.30 |
| *Oenanthe javanica* | Emergent | 40.24 | 2.10 | 0.19 | 2.74 | 191.10 | 7.10 |
| *Phragmites australis* | Emergent | 37.41 | 2.51 | 0.19 | 3.21 | 135.96 | 9.90 |
| *Phragmites australis* | Emergent | 42.31 | 1.62 | 0.10 | 1.99 | 231.26 | 9.50 |
| *Phragmites australis* | Emergent | 40.77 | 1.51 | 0.07 | 6.01 | 179.95 | 9.20 |
| *Phragmites australis* | Emergent | 44.00 | 3.45 | 0.14 | 8.44 | 97.84 | 9.10 |
| *Phragmites australis* | Emergent | 40.44 | 2.74 | 0.13 | 10.37 | -25.75 | 9.00 |
| *Phragmites australis* | Emergent | 42.22 | 3.00 | 0.20 | 4.79 | 172.44 | 8.90 |
| *Phragmites australis* | Emergent | 42.54 | 2.13 | 0.07 | 3.81 | 185.63 | 8.90 |
| *Phragmites australis* | Emergent | 42.65 | 2.23 | 0.07 | 5.34 | 198.63 | 8.90 |
| *Phragmites australis* | Emergent | 41.65 | 1.49 | 0.09 | 10.19 | -22.47 | 8.70 |
| *Phragmites australis* | Emergent | 41.10 | 2.54 | 0.11 | 4.95 | 204.75 | 8.60 |
| *Phragmites australis* | Emergent | 41.60 | 2.59 | 0.09 | 1.47 | 229.85 | 8.60 |
| *Phragmites australis* | Emergent | 40.24 | 1.64 | 0.07 | 6.00 | 180.64 | 8.50 |
| *Phragmites australis* | Emergent | 43.37 | 2.62 | 0.10 | 8.56 | 94.40 | 8.40 |
| *Phragmites australis* | Emergent | 42.08 | 1.61 | 0.06 | 4.92 | 164.73 | 8.40 |
| *Phragmites australis* | Emergent | 42.81 | 1.93 | 0.14 | 4.93 | 187.99 | 8.30 |
| *Phragmites australis* | Emergent | 41.75 | 1.73 | 0.10 | 5.22 | 204.95 | 8.30 |
| *Phragmites australis* | Emergent | 40.92 | 1.62 | 0.07 | 5.22 | 204.95 | 8.30 |
| *Phragmites australis* | Emergent | 44.68 | 2.84 | 0.12 | 9.23 | 140.67 | 8.30 |
| *Phragmites australis* | Emergent | 44.02 | 3.52 | 0.08 | 8.59 | 94.47 | 8.30 |
| *Phragmites australis* | Emergent | 41.13 | 1.80 | 0.08 | 5.20 | 195.20 | 8.20 |
| *Phragmites australis* | Emergent | 42.63 | 2.42 | 0.11 | 8.62 | 93.61 | 8.20 |
| *Phragmites australis* | Emergent | 44.62 | 3.95 | 0.16 | 2.82 | 138.57 | 8.20 |
| *Phragmites australis* | Emergent | 42.36 | 3.59 | 0.12 | 8.71 | 91.59 | 8.20 |
| *Phragmites australis* | Emergent | 43.20 | 3.33 | 0.13 | 4.54 | 213.13 | 8.10 |
| *Phragmites australis* | Emergent | 44.47 | 2.18 | 0.12 | 8.51 | 96.62 | 8.00 |
| *Phragmites australis* | Emergent | 43.94 | 2.63 | 0.12 | 4.96 | 198.32 | 8.00 |
| *Phragmites australis* | Emergent | 39.93 | 2.59 | 0.09 | 9.13 | 50.67 | 8.00 |
| *Phragmites australis* | Emergent | 40.62 | 2.19 | 0.08 | 9.37 | 87.35 | 8.00 |
| *Phragmites australis* | Emergent | 43.45 | 2.72 | 0.07 | 9.96 | 76.25 | 8.00 |
| *Phragmites australis* | Emergent | 41.78 | 1.57 | 0.08 | 5.32 | 187.90 | 7.90 |
| *Phragmites australis* | Emergent | 44.73 | 2.73 | 0.11 | 9.46 | 140.94 | 7.90 |
| *Phragmites australis* | Emergent | 44.29 | 2.57 | 0.10 | 9.48 | 141.17 | 7.90 |
| *Phragmites australis* | Emergent | 43.72 | 2.50 | 0.09 | 8.77 | 91.13 | 7.90 |
| *Phragmites australis* | Emergent | 39.97 | 2.68 | 0.10 | 7.88 | 214.74 | 7.90 |
| *Phragmites australis* | Emergent | 42.75 | 3.46 | 0.12 | 4.14 | 363.56 | 7.90 |
| *Phragmites australis* | Emergent | 42.32 | 2.15 | 0.07 | 3.11 | 213.91 | 7.90 |
| *Phragmites australis* | Emergent | 40.91 | 4.23 | 0.12 | 12.45 | 55.96 | 7.90 |
| *Phragmites australis* | Emergent | 42.24 | 1.84 | 0.15 | 5.27 | 199.71 | 7.80 |
| *Phragmites australis* | Emergent | 43.96 | 3.47 | 0.12 | 7.72 | 131.23 | 7.80 |
| *Phragmites australis* | Emergent | 43.81 | 2.50 | 0.08 | 8.79 | 92.27 | 7.80 |
| *Phragmites australis* | Emergent | 41.64 | 2.39 | 0.07 | 9.60 | 7.90 | 7.80 |
| *Phragmites australis* | Emergent | 41.23 | 2.49 | 0.05 | 8.79 | 92.27 | 7.80 |
| *Phragmites australis* | Emergent | 42.12 | 0.22 | 0.07 | 7.82 | 126.63 | 7.70 |
| *Phragmites australis* | Emergent | 43.38 | 2.68 | 0.30 | 5.16 | 203.43 | 7.70 |
| *Phragmites australis* | Emergent | 44.45 | 2.94 | 0.12 | 8.81 | 90.99 | 7.70 |
| *Phragmites australis* | Emergent | 45.02 | 2.49 | 0.10 | 7.80 | 127.22 | 7.70 |
| *Phragmites australis* | Emergent | 43.52 | 2.26 | 0.09 | 8.78 | 92.73 | 7.70 |
| *Phragmites australis* | Emergent | 43.98 | 2.34 | 0.06 | 13.26 | 43.80 | 7.70 |
| *Phragmites australis* | Emergent | 45.31 | 3.60 | 0.12 | 8.39 | 134.24 | 7.60 |
| *Phragmites australis* | Emergent | 41.36 | 2.34 | 0.06 | 5.52 | 189.48 | 7.60 |
| *Phragmites australis* | Emergent | 42.89 | 2.91 | 0.10 | 8.09 | 105.29 | 7.50 |
| *Phragmites australis* | Emergent | 43.65 | 2.16 | 0.16 | 2.05 | 199.44 | 7.30 |
| *Phragmites australis* | Emergent | 42.52 | 1.59 | 0.08 | 2.74 | 191.10 | 7.10 |
| *Polygonum amphibium* | Floating-leaved | 44.49 | 1.85 | 0.12 | 4.79 | 172.44 | 8.90 |
| *Polygonum amphibium* | Floating-leaved | 45.80 | 3.18 | 0.16 | 9.23 | 140.67 | 8.30 |
| *Polygonum amphibium* | Floating-leaved | 42.47 | 1.61 | 0.10 | 3.11 | 213.91 | 7.90 |
| *Polygonum amphibium* | Floating-leaved | 45.39 | 4.02 | 0.25 | 9.48 | 141.17 | 7.90 |
| *Polygonum hydropiper* | Emergent | 38.87 | 1.39 | 0.08 | 3.21 | 135.96 | 9.90 |
| *Polygonum hydropiper* | Emergent | 43.36 | 2.72 | 0.18 | 2.74 | 191.10 | 7.10 |
| *Polygonum hydropiper* | Emergent | 41.63 | 3.42 | 0.26 | 9.23 | 140.67 | 8.30 |
| *Polygonum hydropiper* | Emergent | 44.54 | 3.41 | 0.11 | 8.51 | 96.80 | 8.10 |
| *Polygonum hydropiper* | Emergent | 41.39 | 2.46 | 0.11 | 9.37 | 87.35 | 8.00 |
| *Polygonum hydropiper* | Emergent | 41.50 | 2.56 | 0.11 | 13.86 | 32.71 | 8.00 |
| *Polygonum hydropiper* | Emergent | 45.29 | 3.46 | 0.12 | 7.88 | 214.74 | 7.90 |
| *Polygonum hydropiper* | Emergent | 43.44 | 3.27 | 0.10 | 9.46 | 140.94 | 7.90 |
| *Polygonum hydropiper* | Emergent | 31.86 | 1.33 | 0.15 | 3.67 | 173.47 | 7.70 |
| *Polygonum hydropiper* | Emergent | 31.68 | 4.09 | 0.31 | 8.15 | 104.01 | 7.60 |
| *Polygonum hydropiper* | Emergent | 43.69 | 3.61 | 0.07 | 8.09 | 105.29 | 7.50 |
| *Polygonum hydropiper* | Emergent | 42.67 | 1.26 | 0.14 | 2.05 | 199.44 | 7.30 |
| *Polygonum hydropiper* | Emergent | 41.61 | 1.03 | 0.10 | 5.34 | 198.63 | 8.90 |
| *Polygonum hydropiper* | Emergent | 41.68 | 2.45 | 0.18 | 4.79 | 172.44 | 8.90 |
| *Polygonum hydropiper* | Emergent | 40.44 | 3.10 | 0.15 | 4.79 | 172.44 | 8.90 |
| *Polygonum hydropiper* | Emergent | 44.78 | 2.63 | 0.05 | 8.62 | 93.61 | 8.20 |
| *Polygonum hydropiper* | Emergent | 45.12 | 2.42 | 0.09 | 4.54 | 213.13 | 8.10 |
| *Polygonum hydropiper* | Emergent | 42.66 | 4.62 | 0.13 | 12.45 | 55.96 | 7.90 |
| *Polygonum hydropiper* | Emergent | 46.53 | 2.65 | 0.08 | 7.80 | 127.22 | 7.70 |
| *Polygonum hydropiper* | Emergent | 44.13 | 2.68 | 0.19 | 8.39 | 134.24 | 7.60 |
| *Polygonum hydropiper* | Emergent | 41.84 | 3.06 | 0.08 | 2.64 | 226.09 | 7.40 |
| *Polygonum hydropiper* | Emergent | 42.59 | 3.38 | 0.22 | -1.31 | 214.46 | 9.90 |
| *Polygonum hydropiper* | Emergent | 42.19 | 2.43 | 0.10 | 6.00 | 180.64 | 8.50 |
| *Potamogeton crispus* | Submerged | 38.38 | 2.31 | 0.16 | 8.62 | 93.61 | 8.20 |
| *Potamogeton crispus* | Submerged | 41.48 | 3.07 | 0.21 | 8.71 | 91.59 | 8.20 |
| *Potamogeton crispus* | Submerged | 41.74 | 3.57 | 0.20 | 9.37 | 87.35 | 8.00 |
| *Potamogeton crispus* | Submerged | 39.18 | 2.90 | 0.24 | 7.88 | 214.74 | 7.90 |
| *Potamogeton crispus* | Submerged | 43.58 | 3.07 | 0.21 | 7.82 | 126.63 | 7.70 |
| *Potamogeton crispus* | Submerged | 40.07 | 2.82 | 0.19 | 3.11 | 213.91 | 7.90 |
| *Potamogeton crispus* | Submerged | 42.80 | 3.38 | 0.29 | 3.67 | 173.47 | 7.70 |
| *Potamogeton crispus* | Submerged | 45.73 | 3.60 | 0.10 | 7.80 | 127.22 | 7.70 |
| *Potamogeton crispus* | Submerged | 34.78 | 1.55 | 0.12 | 3.19 | 136.34 | 9.10 |
| *Potamogeton crispus* | Submerged | 37.03 | 1.82 | 0.14 | 2.83 | 138.42 | 7.70 |
| *Potamogeton cristatus* | Submerged | 37.06 | 2.15 | 0.23 | 4.93 | 187.99 | 8.30 |
| *Potamogeton cristatus* | Submerged | 40.50 | 2.72 | 0.16 | 7.72 | 131.23 | 7.80 |
| *Potamogeton cristatus* | Submerged | 34.90 | 1.53 | 0.09 | 9.60 | 7.90 | 7.80 |
| *Potamogeton cristatus* | Submerged | 38.20 | 2.37 | 0.13 | 8.20 | 107.34 | 7.80 |
| *Potamogeton distinctus* | Floating-leaved | 46.09 | 2.00 | 0.09 | 7.82 | 126.63 | 7.70 |
| *Potamogeton filiformis* | Submerged | 31.30 | 3.16 | 0.25 | 9.60 | 7.90 | 7.80 |
| *Potamogeton heterophyllus* | Submerged | 40.15 | 4.27 | 0.27 | -3.36 | 232.42 | 10.00 |
| *Potamogeton heterophyllus* | Submerged | 41.17 | 1.55 | 0.10 | 5.34 | 198.63 | 8.90 |
| *Potamogeton heterophyllus* | Submerged | 43.35 | 3.29 | 0.17 | 9.37 | 87.35 | 8.00 |
| *Potamogeton heterophyllus* | Submerged | 40.49 | 1.96 | 0.17 | 2.74 | 191.10 | 7.10 |
| *Potamogeton intortifolius* | Submerged | 43.00 | 3.12 | 0.17 | -0.36 | 265.34 | 8.80 |
| *Potamogeton lucens* | Submerged | 38.07 | 1.34 | 0.27 | 8.79 | 92.27 | 7.80 |
| *Potamogeton lucens* | Submerged | 40.96 | 1.47 | 0.09 | 8.81 | 91.62 | 7.80 |
| *Potamogeton lucens* | Submerged | 41.32 | 1.54 | 0.09 | 8.70 | 91.79 | 7.80 |
| *Potamogeton lucens* | Submerged | 40.62 | 1.26 | 0.10 | 8.78 | 92.73 | 7.70 |
| *Potamogeton malaianus* | Submerged | 40.14 | 2.50 | 0.16 | 8.62 | 93.61 | 8.20 |
| *Potamogeton malaianus* | Submerged | 36.96 | 2.93 | 0.14 | 8.51 | 96.80 | 8.10 |
| *Potamogeton malaianus* | Submerged | 41.31 | 1.88 | 0.11 | 8.51 | 96.62 | 8.00 |
| *Potamogeton natans* | Floating-leaved | 41.84 | 2.85 | 0.17 | 9.13 | 50.67 | 8.00 |
| *Potamogeton obtusifolius* | Submerged | 42.29 | 3.65 | 0.26 | -0.36 | 265.34 | 8.80 |
| *Potamogeton obtusifolius* | Submerged | 41.65 | 2.39 | 0.20 | 5.31 | 198.73 | 8.50 |
| *Potamogeton obtusifolius* | Submerged | 41.00 | 3.21 | 0.21 | 7.82 | 126.63 | 7.70 |
| *Potamogeton oxyphyllus* | Submerged | 41.81 | 2.70 | 0.18 | 2.82 | 138.57 | 8.20 |
| *Potamogeton oxyphyllus* | Submerged | 42.72 | 4.23 | 0.30 | 4.14 | 363.56 | 7.90 |
| *Potamogeton pectinatus* | Submerged | 40.14 | 2.68 | 0.18 | -3.36 | 232.42 | 10.00 |
| *Potamogeton pectinatus* | Submerged | 37.26 | 2.23 | 0.16 | 2.32 | 220.94 | 9.60 |
| *Potamogeton pectinatus* | Submerged | 36.70 | 1.60 | 0.19 | 1.99 | 231.26 | 9.50 |
| *Potamogeton pectinatus* | Submerged | 43.29 | 3.06 | 0.20 | -4.02 | 237.59 | 9.50 |
| *Potamogeton pectinatus* | Submerged | 34.44 | 1.85 | 0.18 | 6.01 | 179.95 | 9.20 |
| *Potamogeton pectinatus* | Submerged | 31.61 | 1.19 | 0.07 | 3.19 | 136.34 | 9.10 |
| *Potamogeton pectinatus* | Submerged | 36.92 | 2.81 | 0.21 | 4.79 | 172.44 | 8.90 |
| *Potamogeton pectinatus* | Submerged | 37.54 | 1.28 | 0.11 | 10.19 | -22.47 | 8.70 |
| *Potamogeton pectinatus* | Submerged | 39.54 | 1.67 | 0.14 | 1.47 | 229.85 | 8.60 |
| *Potamogeton pectinatus* | Submerged | 40.99 | 2.87 | 0.16 | 4.44 | 134.98 | 8.60 |
| *Potamogeton pectinatus* | Submerged | 39.23 | 3.59 | 0.29 | -15.12 | 504.81 | 8.50 |
| *Potamogeton pectinatus* | Submerged | 34.49 | 1.73 | 0.09 | 3.41 | 143.42 | 8.50 |
| *Potamogeton pectinatus* | Submerged | 38.53 | 0.69 | 0.05 | 8.54 | 93.99 | 8.40 |
| *Potamogeton pectinatus* | Submerged | 36.56 | 1.10 | 0.12 | 8.64 | 93.61 | 8.30 |
| *Potamogeton pectinatus* | Submerged | 38.88 | 1.50 | 0.14 | 5.22 | 204.95 | 8.30 |
| *Potamogeton pectinatus* | Submerged | 37.01 | 1.30 | 0.10 | 8.64 | 93.34 | 8.30 |
| *Potamogeton pectinatus* | Submerged | 39.14 | 2.22 | 0.20 | 2.82 | 138.57 | 8.20 |
| *Potamogeton pectinatus* | Submerged | 39.69 | 2.53 | 0.22 | 4.85 | 208.27 | 8.20 |
| *Potamogeton pectinatus* | Submerged | 38.47 | 1.51 | 0.11 | 8.62 | 93.61 | 8.20 |
| *Potamogeton pectinatus* | Submerged | 27.96 | 1.68 | 0.12 | 2.82 | 138.57 | 8.20 |
| *Potamogeton pectinatus* | Submerged | 37.63 | 1.53 | 0.11 | 8.71 | 91.59 | 8.20 |
| *Potamogeton pectinatus* | Submerged | 33.85 | 1.39 | 0.09 | 4.02 | 138.27 | 8.20 |
| *Potamogeton pectinatus* | Submerged | 38.35 | 1.71 | 0.12 | 8.51 | 96.80 | 8.10 |
| *Potamogeton pectinatus* | Submerged | 37.65 | 1.89 | 0.12 | 4.96 | 198.32 | 8.00 |
| *Potamogeton pectinatus* | Submerged | 38.01 | 2.44 | 0.14 | 2.95 | 147.12 | 8.00 |
| *Potamogeton pectinatus* | Submerged | 36.33 | 1.35 | 0.07 | 9.37 | 87.35 | 8.00 |
| *Potamogeton pectinatus* | Submerged | 39.50 | 2.10 | 0.10 | 9.96 | 76.25 | 8.00 |
| *Potamogeton pectinatus* | Submerged | 27.49 | 1.30 | 0.06 | 8.98 | 137.45 | 8.00 |
| *Potamogeton pectinatus* | Submerged | 41.12 | 2.02 | 0.09 | 9.13 | 50.67 | 8.00 |
| *Potamogeton pectinatus* | Submerged | 38.53 | 1.17 | 0.21 | 7.88 | 214.74 | 7.90 |
| *Potamogeton pectinatus* | Submerged | 38.21 | 1.20 | 0.13 | 9.46 | 140.94 | 7.90 |
| *Potamogeton pectinatus* | Submerged | 36.82 | 1.45 | 0.11 | 8.66 | 92.14 | 7.90 |
| *Potamogeton pectinatus* | Submerged | 38.48 | 2.17 | 0.13 | 3.11 | 213.91 | 7.90 |
| *Potamogeton pectinatus* | Submerged | 43.82 | 2.12 | 0.11 | 4.14 | 363.56 | 7.90 |
| *Potamogeton pectinatus* | Submerged | 38.83 | 0.94 | 0.09 | 8.79 | 92.27 | 7.80 |
| *Potamogeton pectinatus* | Submerged | 38.37 | 2.23 | 0.14 | 5.27 | 199.71 | 7.80 |
| *Potamogeton pectinatus* | Submerged | 38.77 | 2.03 | 0.21 | 2.83 | 138.42 | 7.70 |
| *Potamogeton pectinatus* | Submerged | 39.34 | 3.31 | 0.26 | 3.67 | 173.47 | 7.70 |
| *Potamogeton pectinatus* | Submerged | 36.67 | 2.26 | 0.17 | 8.15 | 104.01 | 7.60 |
| *Potamogeton pectinatus* | Submerged | 39.69 | 2.44 | 0.13 | 8.09 | 105.29 | 7.50 |
| *Potamogeton perfoliatus* | Submerged | 37.03 | 2.53 | 0.19 | 3.21 | 135.96 | 9.90 |
| *Potamogeton perfoliatus* | Submerged | 36.43 | 1.97 | 0.13 | 2.32 | 220.94 | 9.60 |
| *Potamogeton perfoliatus* | Submerged | 36.54 | 2.22 | 0.17 | 3.19 | 136.34 | 9.10 |
| *Potamogeton perfoliatus* | Submerged | 37.14 | 2.70 | 0.23 | 4.79 | 172.44 | 8.90 |
| *Potamogeton perfoliatus* | Submerged | 39.50 | 0.86 | 0.07 | 5.34 | 198.63 | 8.90 |
| *Potamogeton perfoliatus* | Submerged | 39.84 | 1.90 | 0.20 | 5.22 | 204.95 | 8.30 |
| *Potamogeton perfoliatus* | Submerged | 38.55 | 1.82 | 0.17 | 4.93 | 187.99 | 8.30 |
| *Potamogeton perfoliatus* | Submerged | 37.67 | 1.82 | 0.14 | 8.62 | 93.61 | 8.20 |
| *Potamogeton perfoliatus* | Submerged | 40.79 | 2.95 | 0.21 | 4.54 | 213.13 | 8.10 |
| *Potamogeton perfoliatus* | Submerged | 39.00 | 2.04 | 0.12 | 9.13 | 50.67 | 8.00 |
| *Potamogeton perfoliatus* | Submerged | 36.75 | 3.17 | 0.23 | 9.60 | 7.90 | 7.80 |
| *Potamogeton perfoliatus* | Submerged | 43.08 | 3.94 | 0.20 | 7.72 | 131.23 | 7.80 |
| *Potamogeton perfoliatus* | Submerged | 40.15 | 2.00 | 0.15 | 2.05 | 199.44 | 7.30 |
| *Potamogeton polygonifolius* | Submerged | 35.39 | 2.04 | 0.11 | 8.98 | 137.45 | 8.00 |
| *Potamogeton pusillus* | Submerged | 34.84 | 1.28 | 0.15 | 2.83 | 138.42 | 7.70 |
| *Potamogeton pusillus* | Submerged | 35.71 | 2.25 | 0.19 | 2.32 | 220.94 | 9.60 |
| *Potamogeton pusillus* | Submerged | 39.92 | 1.08 | 0.06 | 5.34 | 198.63 | 8.90 |
| *Potamogeton pusillus* | Submerged | 43.01 | 3.03 | 0.17 | -0.36 | 265.34 | 8.80 |
| *Potamogeton pusillus* | Submerged | 38.77 | 1.96 | 0.24 | 4.85 | 208.27 | 8.20 |
| *Potamogeton pusillus* | Submerged | 39.09 | 1.75 | 0.15 | 4.54 | 213.13 | 8.10 |
| *Potamogeton pusillus* | Submerged | 41.54 | 2.39 | 0.14 | 9.37 | 87.35 | 8.00 |
| *Potamogeton pusillus* | Submerged | 29.06 | 2.16 | 0.16 | 2.05 | 199.44 | 7.30 |
| *Ranunculus chinensis* | Emergent | 41.60 | 0.90 | 0.11 | 2.74 | 191.10 | 7.10 |
| *Ranunculus chinensis* | Emergent | 39.37 | 1.69 | 0.14 | 2.82 | 138.57 | 8.20 |
| *Ranunculus chinensis* | Emergent | 40.90 | 2.41 | 0.10 | 9.13 | 50.67 | 8.00 |
| *Ranunculus chinensis* | Emergent | 42.59 | 2.66 | 0.13 | 2.31 | 141.97 | 7.40 |
| *Ranunculus chinensis* | Emergent | 43.90 | 2.66 | 0.14 | 7.72 | 131.23 | 7.80 |
| *Ranunculus natans* | Floating-leaved | 39.51 | 3.21 | 0.22 | 4.44 | 134.98 | 8.60 |
| *Ranunculus natans* | Floating-leaved | 27.21 | 1.26 | 0.09 | 3.41 | 143.42 | 8.50 |
| *Ranunculus natans* | Floating-leaved | 41.76 | 1.68 | 0.22 | 5.20 | 195.20 | 8.20 |
| *Ranunculus natans* | Floating-leaved | 38.52 | 2.64 | 0.22 | 4.02 | 138.27 | 8.20 |
| *Ranunculus natans* | Floating-leaved | 38.75 | 3.75 | 0.19 | 2.18 | 154.91 | 8.00 |
| *Ranunculus natans* | Floating-leaved | 40.63 | 3.67 | 0.18 | 2.95 | 147.12 | 8.00 |
| *Ranunculus natans* | Floating-leaved | 41.57 | 3.08 | 0.34 | 2.05 | 199.44 | 7.30 |
| *Ranunculus sceleratus* | Emergent | 42.94 | 2.49 | 0.24 | 4.79 | 172.44 | 8.90 |
| *Ranunculus sceleratus* | Emergent | 40.53 | 2.89 | 0.19 | 4.44 | 134.98 | 8.60 |
| *Ranunculus sceleratus* | Emergent | 34.94 | 1.66 | 0.31 | 5.32 | 187.90 | 7.90 |
| *Ranunculus sceleratus* | Emergent | 41.68 | 1.54 | 0.20 | 2.74 | 191.10 | 7.10 |
| *Rumex acetosa* | Emergent | 45.37 | 2.68 | 0.14 | 3.21 | 135.96 | 9.90 |
| *Rumex acetosa* | Emergent | 44.73 | 2.74 | 0.15 | -0.36 | 265.34 | 8.80 |
| *Rumex acetosa* | Emergent | 39.89 | 4.55 | 0.27 | 3.41 | 143.42 | 8.50 |
| *Rumex acetosa* | Emergent | 42.88 | 2.51 | 0.17 | 2.82 | 138.57 | 8.20 |
| *Rumex acetosa* | Emergent | 38.31 | 2.70 | 0.10 | 4.02 | 138.27 | 8.20 |
| *Rumex acetosa* | Emergent | 44.04 | 2.69 | 0.13 | 8.98 | 137.45 | 8.00 |
| *Rumex acetosa* | Emergent | 45.63 | 3.43 | 0.22 | 9.46 | 140.94 | 7.90 |
| *Rumex acetosa* | Emergent | 45.39 | 2.87 | 0.20 | 3.67 | 173.47 | 7.70 |
| *Rumex acetosa* | Emergent | 40.37 | 1.68 | 0.11 | 8.39 | 134.24 | 7.60 |
| *Rumex acetosa* | Emergent | 44.07 | 3.54 | 0.09 | 2.64 | 226.09 | 7.40 |
| *Rumex acetosa* | Emergent | 43.95 | 2.62 | 0.11 | 2.74 | 191.10 | 7.10 |
| *Ruppia maritima* | Submerged | 30.16 | 1.29 | 0.15 | 9.98 | 76.35 | 9.20 |
| *Ruppia maritima* | Submerged | 38.79 | 3.21 | 0.33 | 3.46 | 190.42 | 9.10 |
| *Ruppia maritima* | Submerged | 34.00 | 2.39 | 0.16 | 10.37 | -25.75 | 9.00 |
| *Ruppia maritima* | Submerged | 35.58 | 2.36 | 0.13 | 10.19 | -22.47 | 8.70 |
| *Ruppia maritima* | Submerged | 36.44 | 2.46 | 0.10 | 13.26 | 43.80 | 7.70 |
| *Sagittaria trifolia* | Emergent | 40.65 | 1.03 | 0.26 | 5.34 | 198.63 | 8.90 |
| *Sagittaria trifolia* | Emergent | 42.32 | 3.04 | 0.16 | 8.51 | 96.80 | 8.10 |
| *Scirpus filipes* | Emergent | 45.15 | 1.45 | 0.14 | 9.23 | 140.67 | 8.30 |
| *Scirpus filipes* | Emergent | 45.21 | 1.09 | 0.04 | 8.98 | 137.45 | 8.00 |
| *Scirpus mattfeldianus* | Emergent | 40.52 | 4.13 | 0.17 | 8.71 | 91.59 | 8.20 |
| *Scirpus planiculmis* | Emergent | 43.52 | 0.95 | 0.07 | 3.21 | 135.96 | 9.90 |
| *Scirpus planiculmis* | Emergent | 39.31 | 1.72 | 0.15 | 1.99 | 231.26 | 9.50 |
| *Scirpus planiculmis* | Emergent | 41.43 | 1.22 | 0.09 | 4.95 | 204.75 | 8.60 |
| *Scirpus planiculmis* | Emergent | 41.98 | 1.72 | 0.12 | 5.22 | 204.95 | 8.30 |
| *Scirpus planiculmis* | Emergent | 42.30 | 1.02 | 0.17 | 4.85 | 208.27 | 8.20 |
| *Scirpus planiculmis* | Emergent | 42.39 | 1.52 | 0.09 | 13.86 | 32.71 | 8.00 |
| *Scirpus planiculmis* | Emergent | 40.70 | 1.91 | 0.19 | 9.46 | 140.94 | 7.90 |
| *Scirpus planiculmis* | Emergent | 41.72 | 1.75 | 0.12 | 3.11 | 213.91 | 7.90 |
| *Scirpus planiculmis* | Emergent | 45.60 | 1.19 | 0.09 | 5.27 | 199.71 | 7.80 |
| *Scirpus planiculmis* | Emergent | 42.97 | 0.93 | 0.11 | 5.16 | 203.43 | 7.70 |
| *Scirpus planiculmis* | Emergent | 43.38 | 2.07 | 0.14 | 3.67 | 173.47 | 7.70 |
| *Scirpus planiculmis* | Emergent | 44.78 | 1.19 | 0.08 | 7.80 | 127.22 | 7.70 |
| *Scirpus planiculmis* | Emergent | 42.10 | 0.90 | 0.06 | 5.52 | 189.48 | 7.60 |
| *Scirpus planiculmis* | Emergent | 37.79 | 1.70 | 0.16 | 3.46 | 190.42 | 9.10 |
| *Scirpus planiculmis* | Emergent | 38.65 | 1.70 | 0.13 | 3.81 | 185.63 | 8.90 |
| *Scirpus planiculmis* | Emergent | 39.46 | 1.70 | 0.10 | 10.19 | -22.47 | 8.70 |
| *Scirpus planiculmis* | Emergent | 42.35 | 1.63 | 0.07 | 3.39 | 194.21 | 8.60 |
| *Scirpus planiculmis* | Emergent | 41.78 | 1.59 | 0.13 | 5.20 | 195.20 | 8.20 |
| *Scirpus planiculmis* | Emergent | 41.59 | 2.85 | 0.14 | 9.13 | 50.67 | 8.00 |
| *Scirpus planiculmis* | Emergent | 42.76 | 1.11 | 0.09 | 5.32 | 187.90 | 7.90 |
| *Scirpus planiculmis* | Emergent | 44.78 | 1.17 | 0.07 | 5.31 | 198.90 | 7.90 |
| *Scirpus planiculmis* | Emergent | 44.44 | 1.46 | 0.07 | 8.20 | 107.34 | 7.80 |
| *Scirpus triangulatus* | Emergent | 37.69 | 0.78 | 0.12 | 8.71 | 91.59 | 8.20 |
| *Scirpus triangulatus* | Emergent | 41.92 | 1.42 | 0.09 | 8.51 | 96.80 | 8.10 |
| *Scirpus triangulatus* | Emergent | 40.48 | 1.41 | 0.09 | 8.51 | 96.62 | 8.00 |
| *Scirpus triangulatus* | Emergent | 39.63 | 1.56 | 0.09 | 9.46 | 140.94 | 7.90 |
| *Scirpus triangulatus* | Emergent | 45.00 | 0.81 | 0.05 | 5.27 | 199.71 | 7.80 |
| *Scirpus triangulatus* | Emergent | 43.85 | 2.33 | 0.12 | 7.72 | 131.23 | 7.80 |
| *Scirpus triangulatus* | Emergent | 42.49 | 0.80 | 0.10 | 5.16 | 203.43 | 7.70 |
| *Scirpus triangulatus* | Emergent | 37.51 | 1.71 | 0.07 | 7.82 | 126.63 | 7.70 |
| *Scirpus triangulatus* | Emergent | 39.95 | 1.28 | 0.09 | 8.39 | 134.24 | 7.60 |
| *Scirpus triqueter* | Emergent | 43.20 | 1.47 | 0.08 | 3.21 | 135.96 | 9.90 |
| *Scirpus triqueter* | Emergent | 42.55 | 1.78 | 0.10 | 4.95 | 204.75 | 8.60 |
| *Scirpus triqueter* | Emergent | 40.48 | 2.05 | 0.14 | 7.88 | 214.74 | 7.90 |
| *Scirpus validus* | Emergent | 40.24 | 1.94 | 0.11 | 3.21 | 135.96 | 9.90 |
| *Scirpus validus* | Emergent | 43.24 | 1.91 | 0.10 | 2.32 | 220.94 | 9.60 |
| *Scirpus validus* | Emergent | 42.69 | 0.84 | 0.10 | 8.44 | 97.84 | 9.10 |
| *Scirpus validus* | Emergent | 41.92 | 0.90 | 0.06 | 5.34 | 198.63 | 8.90 |
| *Scirpus validus* | Emergent | 41.59 | 3.04 | 0.15 | 4.79 | 172.44 | 8.90 |
| *Scirpus validus* | Emergent | 40.44 | 3.13 | 0.16 | 4.44 | 134.98 | 8.60 |
| *Scirpus validus* | Emergent | 42.78 | 2.13 | 0.10 | 1.47 | 229.85 | 8.60 |
| *Scirpus validus* | Emergent | 42.28 | 3.11 | 0.08 | 5.31 | 198.73 | 8.50 |
| *Scirpus validus* | Emergent | 39.00 | 2.11 | 0.09 | 8.56 | 94.40 | 8.40 |
| *Scirpus validus* | Emergent | 44.16 | 2.12 | 0.21 | 8.59 | 94.47 | 8.30 |
| *Scirpus validus* | Emergent | 41.98 | 1.74 | 0.11 | 5.22 | 204.95 | 8.30 |
| *Scirpus validus* | Emergent | 43.99 | 1.97 | 0.13 | 9.23 | 140.67 | 8.30 |
| *Scirpus validus* | Emergent | 37.64 | 1.25 | 0.15 | 5.20 | 195.20 | 8.20 |
| *Scirpus validus* | Emergent | 36.47 | 1.29 | 0.11 | 8.62 | 93.61 | 8.20 |
| *Scirpus validus* | Emergent | 42.64 | 1.38 | 0.07 | 4.85 | 208.27 | 8.20 |
| *Scirpus validus* | Emergent | 41.67 | 2.38 | 0.10 | 4.02 | 138.27 | 8.20 |
| *Scirpus validus* | Emergent | 42.90 | 2.24 | 0.09 | 2.82 | 138.57 | 8.20 |
| *Scirpus validus* | Emergent | 42.47 | 2.07 | 0.08 | 4.54 | 213.13 | 8.10 |
| *Scirpus validus* | Emergent | 39.66 | 0.53 | 0.04 | 9.96 | 76.25 | 8.00 |
| *Scirpus validus* | Emergent | 41.86 | 1.86 | 0.11 | 8.98 | 137.45 | 8.00 |
| *Scirpus validus* | Emergent | 39.65 | 2.45 | 0.10 | 4.96 | 198.32 | 8.00 |
| *Scirpus validus* | Emergent | 39.07 | 0.58 | 0.06 | 5.31 | 198.90 | 7.90 |
| *Scirpus validus* | Emergent | 40.72 | 1.43 | 0.10 | 5.32 | 187.90 | 7.90 |
| *Scirpus validus* | Emergent | 40.44 | 2.10 | 0.13 | 9.48 | 141.17 | 7.90 |
| *Scirpus validus* | Emergent | 40.39 | 1.71 | 0.09 | 3.11 | 213.91 | 7.90 |
| *Scirpus validus* | Emergent | 42.01 | 1.49 | 0.07 | 7.88 | 214.74 | 7.90 |
| *Scirpus validus* | Emergent | 43.02 | 1.90 | 0.08 | 5.27 | 199.71 | 7.80 |
| *Scirpus validus* | Emergent | 39.63 | 2.23 | 0.14 | 2.83 | 138.42 | 7.70 |
| *Scirpus validus* | Emergent | 41.03 | 1.60 | 0.09 | 7.82 | 126.63 | 7.70 |
| *Scirpus validus* | Emergent | 42.33 | 1.76 | 0.08 | 8.78 | 92.73 | 7.70 |
| *Scirpus validus* | Emergent | 45.83 | 2.30 | 0.08 | 7.80 | 127.22 | 7.70 |
| *Scirpus validus* | Emergent | 42.16 | 1.18 | 0.09 | 8.39 | 134.24 | 7.60 |
| *Scirpus validus* | Emergent | 43.37 | 2.59 | 0.12 | 8.15 | 104.01 | 7.60 |
| *Scirpus validus* | Emergent | 39.92 | 3.97 | 0.16 | 8.09 | 105.29 | 7.50 |
| *Scirpus validus* | Emergent | 40.29 | 2.48 | 0.14 | 2.05 | 199.44 | 7.30 |
| *Scirpus validus* | Emergent | 42.51 | 1.54 | 0.09 | 2.74 | 191.10 | 7.10 |
| *Sparganium simplex* | Emergent | 41.24 | 4.16 | 0.37 | 2.74 | 191.10 | 7.10 |
| *Sparganium stoloniferum* | Emergent | 41.79 | 1.69 | 0.23 | 3.21 | 135.96 | 9.90 |
| *Sparganium stoloniferum* | Emergent | 39.04 | 1.07 | 0.10 | 3.19 | 136.34 | 9.10 |
| *Sparganium stoloniferum* | Emergent | 40.83 | 4.01 | 0.25 | 8.71 | 91.59 | 8.20 |
| *Sparganium stoloniferum* | Emergent | 42.70 | 1.77 | 0.08 | 4.54 | 213.13 | 8.10 |
| *Sparganium stoloniferum* | Emergent | 41.89 | 2.56 | 0.17 | 8.98 | 137.45 | 8.00 |
| *Sparganium stoloniferum* | Emergent | 40.43 | 1.74 | 0.17 | 9.46 | 140.94 | 7.90 |
| *Sparganium stoloniferum* | Emergent | 39.83 | 1.36 | 0.06 | 9.46 | 140.94 | 7.90 |
| *Sparganium stoloniferum* | Emergent | 43.41 | 2.99 | 0.12 | 8.73 | 91.34 | 7.80 |
| *Sparganium stoloniferum* | Emergent | 40.47 | 2.32 | 0.14 | 8.39 | 134.24 | 7.60 |
| *Sparganium stoloniferum* | Emergent | 42.50 | 3.20 | 0.16 | 8.15 | 104.01 | 7.60 |
| *Sparganium stoloniferum* | Emergent | 36.27 | 1.39 | 0.16 | 2.74 | 191.10 | 7.10 |
| *Triglochin maritimum* | Emergent | 39.78 | 1.85 | 0.13 | 2.32 | 220.94 | 9.60 |
| *Triglochin maritimum* | Emergent | 36.59 | 3.59 | 0.17 | 3.20 | 145.30 | 8.70 |
| *Triglochin maritimum* | Emergent | 36.66 | 2.05 | 0.13 | 4.44 | 134.98 | 8.60 |
| *Triglochin maritimum* | Emergent | 37.14 | 1.67 | 0.10 | 3.11 | 213.91 | 7.90 |
| *Triglochin maritimum* | Emergent | 43.90 | 1.38 | 0.09 | 8.20 | 107.34 | 7.80 |
| *Triglochin palustre* | Emergent | 42.39 | 2.45 | 0.12 | -1.31 | 214.46 | 9.90 |
| *Triglochin palustre* | Emergent | 37.14 | 2.96 | 0.15 | 1.56 | 231.08 | 9.70 |
| *Triglochin palustre* | Emergent | 37.10 | 2.66 | 0.19 | 2.32 | 220.94 | 9.60 |
| *Triglochin palustre* | Emergent | 26.19 | 1.23 | 0.08 | 1.99 | 231.26 | 9.50 |
| *Triglochin palustre* | Emergent | 42.11 | 0.90 | 0.12 | 4.79 | 172.44 | 8.90 |
| *Triglochin palustre* | Emergent | 40.45 | 1.89 | 0.17 | -0.36 | 265.34 | 8.80 |
| *Triglochin palustre* | Emergent | 36.60 | 2.13 | 0.12 | 1.47 | 229.85 | 8.60 |
| *Triglochin palustre* | Emergent | 36.17 | 1.81 | 0.09 | 3.39 | 194.21 | 8.60 |
| *Triglochin palustre* | Emergent | 38.28 | 1.66 | 0.13 | -15.12 | 504.81 | 8.50 |
| *Triglochin palustre* | Emergent | 35.65 | 2.27 | 0.13 | 3.41 | 143.42 | 8.50 |
| *Triglochin palustre* | Emergent | 42.86 | 0.78 | 0.09 | 5.22 | 204.95 | 8.30 |
| *Triglochin palustre* | Emergent | 38.05 | 2.68 | 0.19 | 4.02 | 138.27 | 8.20 |
| *Triglochin palustre* | Emergent | 35.26 | 3.21 | 0.22 | 4.85 | 208.27 | 8.20 |
| *Triglochin palustre* | Emergent | 41.32 | 2.28 | 0.11 | 2.82 | 138.57 | 8.20 |
| *Triglochin palustre* | Emergent | 40.30 | 3.90 | 0.16 | 5.20 | 195.20 | 8.20 |
| *Triglochin palustre* | Emergent | 39.11 | 1.37 | 0.11 | 8.98 | 137.45 | 8.00 |
| *Triglochin palustre* | Emergent | 36.55 | 1.99 | 0.13 | 4.96 | 198.32 | 8.00 |
| *Triglochin palustre* | Emergent | 38.53 | 1.64 | 0.06 | 9.13 | 50.67 | 8.00 |
| *Triglochin palustre* | Emergent | 42.79 | 1.22 | 0.11 | 9.46 | 140.94 | 7.90 |
| *Triglochin palustre* | Emergent | 39.58 | 2.84 | 0.10 | 4.14 | 363.56 | 7.90 |
| *Triglochin palustre* | Emergent | 32.50 | 1.88 | 0.17 | 8.20 | 107.34 | 7.80 |
| *Triglochin palustre* | Emergent | 40.56 | 1.92 | 0.12 | 2.83 | 138.42 | 7.70 |
| *Triglochin palustre* | Emergent | 37.92 | 1.64 | 0.10 | 3.67 | 173.47 | 7.70 |
| *Triglochin palustre* | Emergent | 43.53 | 1.13 | 0.06 | 5.16 | 203.43 | 7.70 |
| *Triglochin palustre* | Emergent | 39.20 | 1.85 | 0.10 | 8.39 | 134.24 | 7.60 |
| *Triglochin palustre* | Emergent | 43.31 | 1.43 | 0.09 | 2.31 | 141.97 | 7.40 |
| *Triglochin palustre* | Emergent | 44.81 | 1.11 | 0.11 | 2.05 | 199.44 | 7.30 |
| *Typha angustifolia* | Emergent | 46.19 | 1.28 | 0.10 | 8.44 | 97.84 | 9.10 |
| *Typha angustifolia* | Emergent | 44.75 | 2.82 | 0.08 | 8.56 | 94.40 | 8.40 |
| *Typha angustifolia* | Emergent | 35.15 | 1.69 | 0.20 | 5.20 | 195.20 | 8.20 |
| *Typha angustifolia* | Emergent | 43.81 | 2.90 | 0.15 | 8.62 | 93.61 | 8.20 |
| *Typha angustifolia* | Emergent | 44.98 | 1.65 | 0.07 | 8.71 | 91.59 | 8.20 |
| *Typha angustifolia* | Emergent | 45.98 | 1.43 | 0.10 | 5.32 | 187.90 | 7.90 |
| *Typha angustifolia* | Emergent | 44.26 | 1.18 | 0.07 | 7.88 | 214.74 | 7.90 |
| *Typha angustifolia* | Emergent | 47.17 | 1.95 | 0.11 | 9.46 | 140.94 | 7.90 |
| *Typha angustifolia* | Emergent | 43.95 | 0.48 | 0.12 | 7.72 | 131.23 | 7.80 |
| *Typha angustifolia* | Emergent | 45.15 | 1.77 | 0.08 | 8.15 | 104.01 | 7.60 |
| *Typha angustifolia* | Emergent | 43.74 | 1.37 | 0.10 | 2.74 | 191.10 | 7.10 |
| *Typha angustifolia* | Emergent | 44.15 | 1.36 | 0.08 | 7.82 | 126.63 | 7.70 |
| *Typha davidiana* | Emergent | 46.06 | 2.20 | 0.12 | 1.47 | 229.85 | 8.60 |
| *Typha davidiana* | Emergent | 44.89 | 1.08 | 0.09 | 5.22 | 204.95 | 8.30 |
| *Typha davidiana* | Emergent | 41.81 | 1.57 | 0.08 | 5.20 | 195.20 | 8.20 |
| *Typha davidiana* | Emergent | 45.50 | 1.39 | 0.08 | 4.54 | 213.13 | 8.10 |
| *Typha davidiana* | Emergent | 42.98 | 2.08 | 0.21 | 7.80 | 127.22 | 7.70 |
| *Typha davidiana* | Emergent | 44.63 | 1.69 | 0.09 | 8.39 | 134.24 | 7.60 |
| *Typha gracilis* | Emergent | 44.23 | 2.09 | 0.13 | 1.47 | 229.85 | 8.60 |
| *Typha gracilis* | Emergent | 42.63 | 1.67 | 0.14 | 4.85 | 208.27 | 8.20 |
| *Typha gracilis* | Emergent | 45.08 | 1.78 | 0.06 | 8.51 | 96.80 | 8.10 |
| *Typha gracilis* | Emergent | 42.60 | 1.25 | 0.19 | 9.96 | 76.25 | 8.00 |
| *Typha gracilis* | Emergent | 45.94 | 1.03 | 0.04 | 8.98 | 137.45 | 8.00 |
| *Typha gracilis* | Emergent | 45.56 | 1.00 | 0.10 | 9.46 | 140.94 | 7.90 |
| *Typha gracilis* | Emergent | 42.89 | 1.42 | 0.13 | 3.11 | 213.91 | 7.90 |
| *Typha gracilis* | Emergent | 44.66 | 1.11 | 0.07 | 5.27 | 199.71 | 7.80 |
| *Typha gracilis* | Emergent | 42.01 | 1.28 | 0.10 | 5.16 | 203.43 | 7.70 |
| *Typha gracilis* | Emergent | 43.43 | 1.64 | 0.10 | 3.67 | 173.47 | 7.70 |
| *Typha gracilis* | Emergent | 41.33 | 1.14 | 0.09 | 5.52 | 189.48 | 7.60 |
| *Typha gracilis* | Emergent | 44.98 | 2.13 | 0.14 | 8.15 | 104.01 | 7.60 |
| *Typha gracilis* | Emergent | 45.31 | 1.83 | 0.06 | 8.39 | 134.24 | 7.60 |
| *Typha gracilis* | Emergent | 42.63 | 2.20 | 0.16 | 8.09 | 105.29 | 7.50 |
| *Typha minima* | Emergent | 44.93 | 0.24 | 0.04 | 5.34 | 198.63 | 8.90 |
| *Typha minima* | Emergent | 40.51 | 2.55 | 0.13 | 4.14 | 363.56 | 7.90 |
| *Typha minima* | Emergent | 45.77 | 1.45 | 0.07 | 5.27 | 199.71 | 7.80 |
| *Typha minima* | Emergent | 43.97 | 0.89 | 0.07 | 7.82 | 126.63 | 7.70 |
| *Typha minima* | Emergent | 43.86 | 1.07 | 0.06 | 5.52 | 189.48 | 7.60 |
| *Typha minima* | Emergent | 44.08 | 1.13 | 0.44 | 2.05 | 199.44 | 7.30 |
| *Typha orientalis* | Emergent | 43.76 | 1.86 | 0.10 | 2.32 | 220.94 | 9.60 |
| *Typha orientalis* | Emergent | 43.23 | 2.22 | 0.13 | 3.81 | 185.63 | 8.90 |
| *Typha orientalis* | Emergent | 43.40 | 2.32 | 0.15 | 3.39 | 194.21 | 8.60 |
| *Typha orientalis* | Emergent | 41.71 | 1.83 | 0.09 | 4.95 | 204.75 | 8.60 |
| *Typha orientalis* | Emergent | 38.86 | 3.59 | 0.23 | 4.92 | 164.73 | 8.40 |
| *Typha orientalis* | Emergent | 39.34 | 2.62 | 0.23 | 4.93 | 187.99 | 8.30 |
| *Typha orientalis* | Emergent | 43.78 | 1.53 | 0.13 | 9.23 | 140.67 | 8.30 |
| *Typha orientalis* | Emergent | 43.16 | 2.29 | 0.13 | 8.59 | 94.47 | 8.30 |
| *Typha orientalis* | Emergent | 42.60 | 1.34 | 0.17 | 9.13 | 50.67 | 8.00 |
| *Typha orientalis* | Emergent | 44.37 | 0.80 | 0.06 | 8.51 | 96.62 | 8.00 |
| *Typha orientalis* | Emergent | 47.79 | 1.09 | 0.08 | 8.77 | 91.13 | 7.90 |
| *Typha orientalis* | Emergent | 45.00 | 2.42 | 0.10 | 9.48 | 141.17 | 7.90 |
| *Typha orientalis* | Emergent | 42.78 | 1.17 | 0.08 | 2.83 | 138.42 | 7.70 |
| *Typha orientalis* | Emergent | 41.07 | 1.33 | 0.04 | 13.26 | 43.80 | 7.70 |
| *Typha orientalis* | Emergent | 42.14 | 1.35 | 0.11 | 8.39 | 134.24 | 7.60 |
| *Typha orientalis* | Emergent | 40.32 | 2.85 | 0.16 | 2.31 | 141.97 | 7.40 |
| *Typha pallida* | Emergent | 41.24 | 2.50 | 0.25 | 1.56 | 231.08 | 9.70 |
| *Typha pallida* | Emergent | 42.96 | 2.03 | 0.12 | 2.32 | 220.94 | 9.60 |
| *Typha pallida* | Emergent | 41.68 | 1.33 | 0.10 | 1.99 | 231.26 | 9.50 |
| *Typha pallida* | Emergent | 45.87 | 1.95 | 0.12 | 13.86 | 32.71 | 8.00 |
| *Typha pallida* | Emergent | 42.27 | 2.70 | 0.16 | 7.01 | 118.71 | 7.80 |
| *Typha pallida* | Emergent | 45.42 | 1.94 | 0.08 | 7.80 | 127.22 | 7.70 |
| *Typha pallida* | Emergent | 44.32 | 2.07 | 0.10 | 2.05 | 199.44 | 7.30 |
| *Typha przewalskii* | Emergent | 45.61 | 1.17 | 0.25 | 3.21 | 135.96 | 9.90 |
| *Typha przewalskii* | Emergent | 42.39 | 1.75 | 0.09 | 9.60 | 7.90 | 7.80 |
| *Typha przewalskii* | Emergent | 43.18 | 1.89 | 0.09 | 7.01 | 118.71 | 7.80 |
| *Utricularia aurea* | Submerged | 40.45 | 2.10 | 0.10 | 5.31 | 198.90 | 7.90 |
| *Utricularia intermedia* | Submerged | 26.88 | 1.39 | 0.10 | 2.32 | 220.94 | 9.60 |
| *Utricularia intermedia* | Submerged | 32.61 | 2.75 | 0.14 | 13.86 | 32.71 | 8.00 |
| *Utricularia minor* | Submerged | 31.13 | 1.40 | 0.07 | 8.78 | 92.73 | 7.70 |
| *Utricularia vulgaris* | Submerged | 43.45 | 1.02 | 0.09 | 6.00 | 180.64 | 8.50 |
| *Utricularia vulgaris* | Submerged | 32.27 | 2.14 | 0.14 | 4.54 | 213.13 | 8.10 |
| *Utricularia vulgaris* | Submerged | 29.60 | 1.30 | 0.19 | 9.46 | 140.94 | 7.90 |
| *Utricularia vulgaris* | Submerged | 35.88 | 1.08 | 0.08 | 8.79 | 92.27 | 7.80 |
| *Utricularia vulgaris* | Submerged | 38.59 | 1.17 | 0.08 | 8.81 | 91.13 | 7.80 |
| *Utricularia vulgaris* | Submerged | 39.35 | 0.92 | 0.05 | 8.81 | 91.62 | 7.80 |
| *Utricularia vulgaris* | Submerged | 39.70 | 2.01 | 0.05 | 8.79 | 91.11 | 7.80 |
| *Utricularia vulgaris* | Submerged | 31.74 | 1.56 | 0.18 | 5.16 | 203.43 | 7.70 |
| *Utricularia vulgaris* | Submerged | 31.52 | 1.34 | 0.08 | 8.81 | 90.99 | 7.70 |
| *Utricularia vulgaris* | Submerged | 38.24 | 1.40 | 0.07 | 8.74 | 91.48 | 7.70 |
| *Utricularia vulgaris* | Submerged | 29.73 | 1.40 | 0.07 | 2.83 | 138.42 | 7.70 |
| *Utricularia vulgaris* | Submerged | 31.45 | 1.64 | 0.06 | 8.78 | 92.73 | 7.70 |
| *Utricularia vulgaris* | Submerged | 38.76 | 2.44 | 0.23 | 2.05 | 199.44 | 7.30 |
| *Veronica anagallis-aquatica* | Emergent | 43.08 | 4.30 | 0.16 | 4.44 | 134.98 | 8.60 |
| *Veronica anagallis-aquatica* | Emergent | 45.98 | 2.77 | 0.11 | 4.85 | 208.27 | 8.20 |
| *Veronica anagallis-aquatica* | Emergent | 37.17 | 5.10 | 0.26 | 12.45 | 55.96 | 7.90 |
| *Veronica anagallis-aquatica* | Emergent | 39.48 | 2.28 | 0.11 | 5.31 | 198.90 | 7.90 |
| *Veronica anagallis-aquatica* | Emergent | 45.15 | 4.34 | 0.12 | 4.14 | 363.56 | 7.90 |
| *Veronica anagallis-aquatica* | Emergent | 44.68 | 3.85 | 0.19 | 3.67 | 173.47 | 7.70 |
| *Veronica anagallis-aquatica* | Emergent | 45.36 | 4.06 | 0.10 | 7.80 | 127.22 | 7.70 |
| *Veronica anagallis-aquatica* | Emergent | 43.65 | 2.57 | 0.18 | 5.52 | 189.48 | 7.60 |
| *Veronica anagallis-aquatica* | Emergent | 43.72 | 1.22 | 0.19 | 2.74 | 191.10 | 7.10 |
| *Veronica beccabunga* | Emergent | 34.46 | 2.10 | 0.07 | 3.19 | 136.34 | 9.10 |
| *Veronica undulata* | Emergent | 36.17 | 3.23 | 0.24 | 2.32 | 220.94 | 9.60 |
| *Veronica undulata* | Emergent | 39.27 | 3.30 | 0.16 | 4.54 | 213.13 | 8.10 |
| *Veronica undulata* | Emergent | 47.04 | 2.83 | 0.23 | 4.96 | 198.32 | 8.00 |
| *Veronica undulata* | Emergent | 37.17 | 3.21 | 0.18 | 5.16 | 203.43 | 7.70 |
| *Veronica undulata* | Emergent | 44.94 | 3.07 | 0.08 | 7.82 | 126.63 | 7.70 |
| *Veronica undulata* | Emergent | 39.36 | 4.89 | 0.24 | 2.64 | 226.09 | 7.40 |
| *Zannichellia palustris* | Submerged | 40.84 | 3.70 | 0.30 | -1.31 | 214.46 | 9.90 |
| *Zannichellia palustris* | Submerged | 43.54 | 3.06 | 0.25 | 1.56 | 231.08 | 9.70 |
| *Zannichellia palustris* | Submerged | 41.12 | 2.02 | 0.20 | 1.99 | 231.26 | 9.50 |
| *Zannichellia palustris* | Submerged | 36.52 | 2.57 | 0.18 | 10.37 | -25.75 | 9.00 |
| *Zannichellia palustris* | Submerged | 32.06 | 1.48 | 0.12 | 3.81 | 185.63 | 8.90 |
| *Zannichellia palustris* | Submerged | 41.01 | 2.18 | 0.22 | 1.47 | 229.85 | 8.60 |
| *Zannichellia palustris* | Submerged | 39.13 | 2.98 | 0.23 | 4.44 | 134.98 | 8.60 |
| *Zannichellia palustris* | Submerged | 38.49 | 2.78 | 0.16 | 3.39 | 194.21 | 8.60 |
| *Zannichellia palustris* | Submerged | 41.46 | 2.69 | 0.22 | 3.41 | 143.42 | 8.50 |
| *Zannichellia palustris* | Submerged | 37.28 | 1.49 | 0.16 | 4.92 | 164.73 | 8.40 |
| *Zannichellia palustris* | Submerged | 40.73 | 3.04 | 0.18 | 4.92 | 164.73 | 8.40 |
| *Zannichellia palustris* | Submerged | 37.82 | 2.34 | 0.28 | 2.82 | 138.57 | 8.20 |
| *Zannichellia palustris* | Submerged | 35.56 | 2.23 | 0.21 | 4.02 | 138.27 | 8.20 |
| *Zannichellia palustris* | Submerged | 41.43 | 3.20 | 0.22 | 2.95 | 147.12 | 8.00 |
| *Zannichellia palustris* | Submerged | 40.48 | 2.60 | 0.13 | 9.13 | 50.67 | 8.00 |
| *Zannichellia palustris* | Submerged | 39.81 | 2.78 | 0.17 | 7.82 | 126.63 | 7.70 |
| *Zannichellia palustris* | Submerged | 38.37 | 1.79 | 0.10 | 2.83 | 138.42 | 7.70 |
| *Zannichellia palustris* | Submerged | 39.00 | 2.41 | 0.12 | 7.80 | 127.22 | 7.70 |

**Appendix S2** RDA based on environmental factors and leaf traits. Environmental factors: Alt, altitude; Long, longitude; Lat, latitude; pH; MAT, mean annual temperature and MAP, mean annual precipitation. Leaf stoichiometry traits: leaf C%, N%, P% and C:N, C:P and N:P ratios.


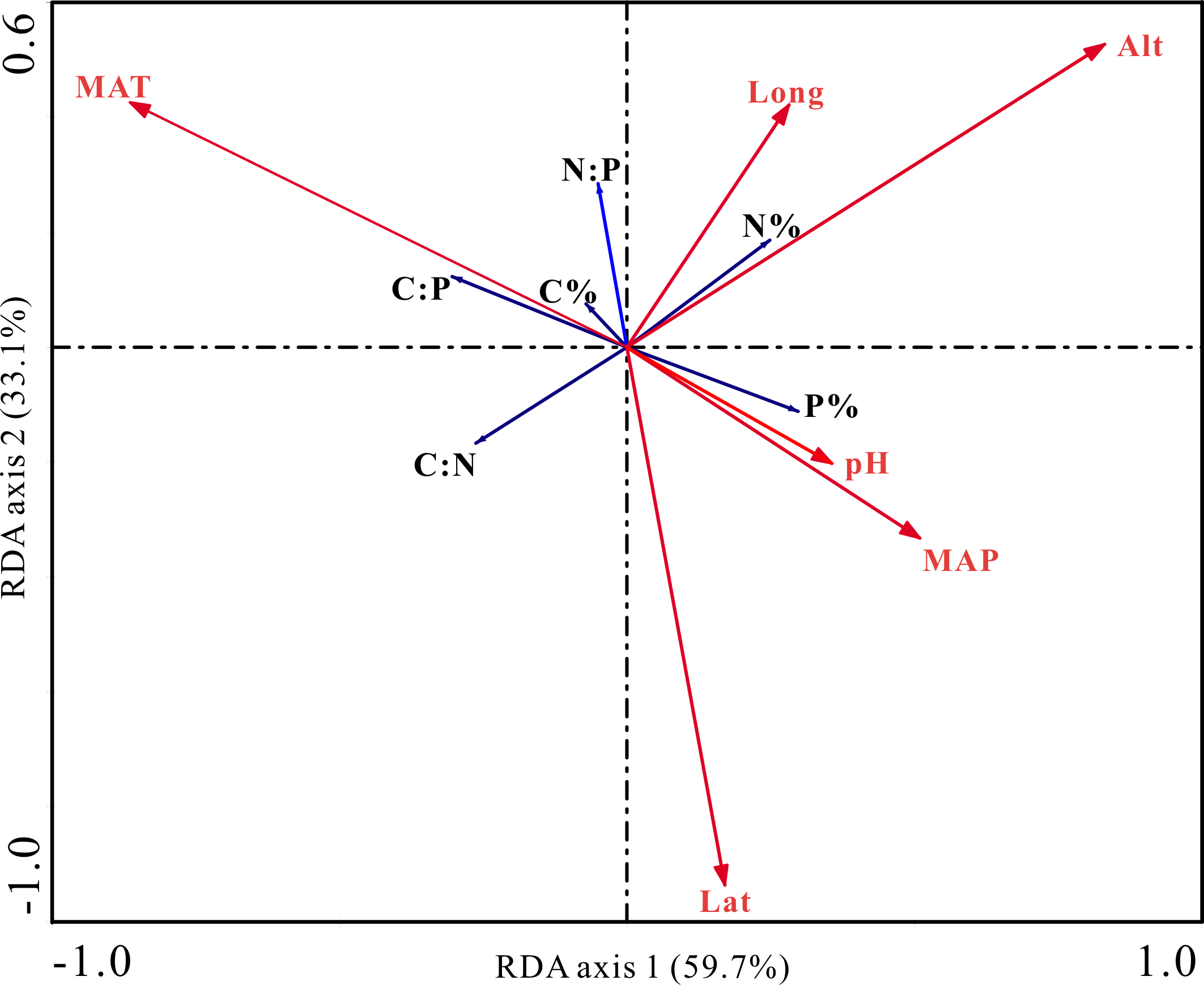


**Appendix S3** Pearson correlation among parameters.

|  | longitude | latitude | altitude | MAT | MAP | pH |
| --- | --- | --- | --- | --- | --- | --- |
| longitude | 1.00 | -0.526^**^ | 0.474^**^ | -0.027 | -0.007 | -0.105^**^ |
| latitude |  | 1.00 | -0.378^**^ | -0.604^**^ | 0.577^**^ | 0.232^**^ |
| altitude |  |  | 1.00 | -0.494^**^ | 0.187^**^ | 0.187^**^ |
| MAT |  |  |  | 1.00 | -0.769^**^ | -0.351^**^ |
| MAP |  |  |  |  | 1.00 | 0.192^**^ |
| pH |  |  |  |  |  | 1.00 |

*^**^p < 0.01, ^*^p < 0.05*

**Appendix S4** The mainly influencing environmental factors of leaf stoichiometry pattern of aquatic macrophytes among the three climatic zones in China.

| Collection area | Mainly environmental factors | Influence | Reference |
| --- | --- | --- | --- |
| The Tibetan plateau | Temperature (leaf N%) | Negative | Wang et al., 2015 |
|  | Temperature (leaf P%) | Negative |  |
| Eastern China | Temperature (leaf N%) | Positive | Xia et al., 2014 |
|  | Temperature (leaf P%) | Negative |  |
| Northwestern China | pH (leaf C%) | Negative | In our study |
|  | Temperature (leaf N%) | Negative |  |
|  | Temperature (leaf P%) | Negative |  |

Wang, Z., Xia, C. X., Yu, D., and Wu, Z. G. (2015). Low-temperature induced leaf elements accumulation in aquatic macrophytes across Tibetan Plateau. Ecol. Eng. 75, 1-8. doi: 10.1093/chromsci/bmu209

Xia, C. X., Yu, D., Wang, Z., and Xie, D. (2014). Stoichiometry patterns of leaf carbon,nitrogen and phosphorous in aquatic macrophytes in eastern China. Ecol. Eng. 70, 406-413. doi: 10.1016/j.ecoleng.2014.06.018
